# Effectiveness of Online vs In-Person Care for Adults With Psoriasis: A Randomized Clinical Trial

**DOI:** 10.1001/jamanetworkopen.2018.3062

**Published:** 2018-10-05

**Authors:** April W. Armstrong, Cindy J. Chambers, Emanual Maverakis, Michelle Y. Cheng, Cory A. Dunnick, Mary-Margaret Chren, Joel M. Gelfand, David J. Wong, Brittany M. Gibbons, Caitlin M. Gibbons, Josefina Torres, Andrea C. Steel, Elizabeth A. Wang, Caitlin M. Clark, Sanminder Singh, Heather A. Kornmehl, Reason Wilken, Aleksandra G. Florek, Adam R. Ford, Chelsea Ma, Nazanin Ehsani-Chimeh, Sucharita Boddu, Mayumi Fujita, Paulina M. Young, Cesar Rivas-Sanchez, Brenda I. Cornejo, Laura C. Serna, Eric R. Carlson, Christianne J. Lane

**Affiliations:** 1 Department of Dermatology, Keck School of Medicine of the University of Southern California, Los Angeles; 2 Department of Dermatology, University of California Davis School of Medicine, Sacramento; 3 Department of Dermatology, University of Colorado Denver, Anschutz Medical Campus, Aurora; 4 Department of Dermatology, Vanderbilt University Medical Center, Nashville, Tennessee; 5 Department of Dermatology, University of Pennsylvania Perelman School of Medicine, Philadelphia; 6 Department of Dermatology, Stanford University School of Medicine, Redwood City, California; 7 University of Hawaii–Manoa John A. Burns School of Medicine, Honolulu; 8 Drexel University College of Medicine, Philadelphia, Pennsylvania; 9 Clinical and Translational Science Institute, Keck School of Medicine of the University of Southern California, Los Angeles; 10 Rocky Vista University College of Osteopathic Medicine, Parker, Colorado; 11 Department of Preventive Medicine, Keck School of Medicine of the University of Southern California, Los Angeles

## Abstract

**Importance:**

Innovative, online models of specialty-care delivery are critical to improving patient access and outcomes.

**Objective:**

To determine whether an online, collaborative connected-health model results in equivalent clinical improvements in psoriasis compared with in-person care.

**Design, Setting, and Participants:**

The Patient-Centered Outcomes Research Institute Psoriasis Teledermatology Trial is a 12-month, pragmatic, randomized clinical equivalency trial to evaluate the effect of an online model for psoriasis compared with in-person care. Participant recruitment and study visits took place at multicenter ambulatory clinics from February 2, 2015, to August 18, 2017. Participants were adults with psoriasis in Northern California, Southern California, and Colorado. The eligibility criteria were an age of 18 years or older, having physician-diagnosed psoriasis, access to the internet and a digital camera or mobile phone with a camera, and having a primary care physician. Analyses were on an intention-to-treat basis.

**Interventions:**

Participants were randomized 1:1 to receive online or in-person care (148 randomized to online care and 148 randomized to in-person care). The online model enabled patients and primary care physicians to access dermatologists online asynchronously. The dermatologists provided assessments, recommendations, education, and prescriptions online. The in-person group sought care in person. The frequency of online or in-person visits was determined by medical necessity. All participants were exposed to their respective interventions for 12 months.

**Main Outcomes and Measures:**

The prespecified primary outcome was the difference in improvement in the self-administered Psoriasis Area and Severity Index (PASI) score between the online and in-person groups. Prespecified secondary outcomes included body surface area (BSA) affected by psoriasis and the patient global assessment score.

**Results:**

Of the 296 randomized participants, 147 were women, 149 were men, 187 were white, and the mean (SD) age was 49 (14) years. The adjusted difference between the online and in-person groups in the mean change in the self-administered PASI score during the 12-month study period was –0.27 (95% CI, –0.85 to 0.31). The difference in the mean change in BSA affected by psoriasis between the 2 groups was –0.05% (95% CI, –1.58% to 1.48%). Between-group differences in the PASI score and BSA were within prespecified equivalence margins, which demonstrated equivalence between the 2 interventions. The difference in the mean change in the patient global assessment score between the 2 groups was –0.11 (95% CI, –0.32 to 0.10), which exceeded the equivalence margin, with the online group displaying greater improvement.

**Conclusions and Relevance:**

The online, collaborative connected-health model was as effective as in-person management in improving clinical outcomes among patients with psoriasis. Innovative telehealth delivery models that emphasize collaboration, quality, and efficiency can be transformative to improving patient-centered outcomes in chronic diseases.

**Trial Registration:**

ClinicalTrials.gov Identifier: NCT02358135

## Key Points

**Question:**

Does an online, collaborative connected-health delivery model result in equivalent improvements in disease severity compared with in-person care among patients with psoriasis?

**Findings:**

In a 12-month randomized clinical equivalency trial, adults with psoriasis randomized to the online model experienced improvement in disease severity equivalent to those randomized to in-person management. Differences between the 2 arms were within prespecified equivalence margins.

**Meaning:**

Innovative telehealth models can play a critical role in the effective management of chronic skin diseases.

## Teaser

This randomized clinical trial examines whether an online, collaborative connected-health model results in equivalent clinical improvements compared with in-person care among US adults with psoriasis.

## Introduction

Skin diseases account for 30% of all physician office visits.^1,2^ Chronic skin diseases are associated with markedly decreased quality of life and financial consequences.^3^ In many parts of the world, patients lack access to dermatologists, especially patients in rural and underserved communities.^4,5,6,7^ In the United States, even after initial evaluation by dermatologists, patients in remote or underserved areas have difficulties maintaining regular access to dermatologists for follow-up care.^4,7^ Consequently, many patients with chronic skin diseases, such as psoriasis, lack regular specialty care and experience poorer clinical outcomes and reduced quality of life.^8^

Psoriasis is a chronic, inflammatory skin disease that affects approximately 3% of the US population.^9,10^ Psoriasis manifests as thick, red, scaly plaques that can occur anywhere on the body and are associated with itching, pain, and bleeding. The treatment of psoriasis is complex and involves long-term monitoring.^11^ In addition, psoriasis is associated with a number of serious comorbidities, including inflammatory arthritis, cardiovascular disease, and severe depression.^12,13^ Psoriasis is independently associated with approximately 11 500 annual events of myocardial infarction, stroke, and cardiovascular deaths.^14,15^ Thus, a team-based approach in which dermatologists and primary care physicians (PCPs) comanage psoriasis and its comorbidities is critical to improving the overall well-being of patients with psoriasis.

Connected health is a model for health care delivery that uses technology to provide health care remotely. Teledermatology is a type of connected health in which remote diagnosis and treatment of patients’ skin diseases occur by means of telecommunications technology.^16,17^ The application of teledermatology has met with varied success.^18^ Although ample evidence supports the diagnostic accuracy and reliability of asynchronous teledermatology,^19,20^ traditional consultative teledermatology has not been as widely adopted as previously expected.^21,22^ Real-world challenges with traditional asynchronous teledermatology include a lack of collaborative and informed communication among patients, PCPs, and specialists.

In this study, we evaluated an innovative, collaborative connected-health model in which patients and PCPs can access dermatologists online directly and asynchronously via a pragmatic trial. The primary aim of this pragmatic trial was to determine whether this online, collaborative connected-health model results in equivalent improvements in psoriasis disease severity compared with in-person care.

## Methods

### Study Design

**Figure 1.**
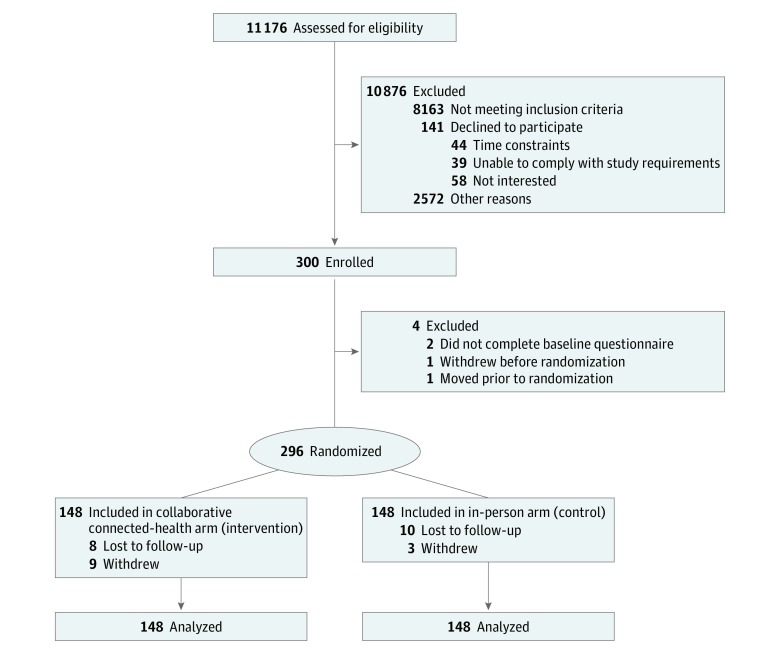
CONSORT Flow Diagram

**Figure 2.**
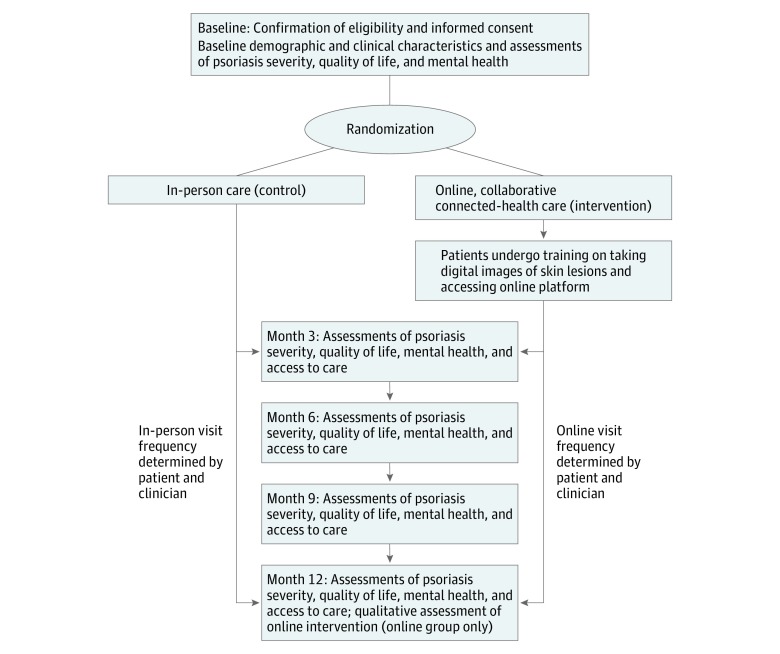
Overview of Pragmatic Randomized Clinical Trial Comparing Online vs In-Person Care in Patients With Psoriasis

The Patient-Centered Outcomes Research Institute Psoriasis Teledermatology Trial is a 12-month, pragmatic, randomized clinical equivalency trial with a parallel-group design that evaluated the effect of an online, collaborative connected-health model for psoriasis management compared with in-person care (Figure 1 and Figure 2) (the study protocol is available in the Supplement). This multicenter study was conducted at community clinics and outpatient clinics affiliated with the University of Southern California; the University of California, Davis; and the University of Colorado. This study was approved by the University of Southern California; University of California, Davis; and University of Colorado institutional review boards. All patients provided written informed consent and were compensated nominally for their participation. This study followed the Consolidated Standards of Reporting Trials (CONSORT) reporting guideline for clinical trials as well as the CONSORT extensions for pragmatic trials and equivalence trials.^23^

### Participants

We recruited participants from the outpatient clinics and the general adult populations in Northern California, Southern California, and Colorado. Specifically, we recruited patients from practice-based research networks, federally qualified health centers, and university-based clinics in Colorado and California. We also recruited from patients affiliated with the National Psoriasis Foundation and the general public.

The eligibility criteria were an age of 18 years or older, having physician-diagnosed plaque psoriasis (new or previous diagnosis), access to the internet and a digital camera or a mobile phone with camera features, and having a PCP or the ability to establish primary care. No changes were made to the eligibility criteria after trial commencement. We collected demographic information from patients, including age, sex, race/ethnicity, marital status, educational level, and working status (Table 1).

**Table 1.** Baseline Patient Demographic and Clinical Characteristics

Characteristics	Patients, No. (%)		
	Online Group (n = 148)	In-Person Group (n = 148)	Total (N = 296)
Sex			
Male	75 (50.7)	74 (50.0)	149 (50.3)
Female	73 (49.3)	74 (50.0)	147 (49.7)
Race^a^^,^^b^			
American Indian or Alaska Native	3 (2.0)	2 (1.4)	5 (1.7)
Asian	13 (8.8)	6 (4.1)	19 (6.4)
Black or African American	5 (3.4)	3 (2.0)	8 (2.7)
Pacific Islander	3 (2.0)	2 (1.4)	5 (1.7)
White	90 (60.8)	97 (65.5)	187 (63.2)
Other	36 (24.3)	36 (24.3)	72 (24.3)
Ethnicity			
Hispanic or Latino	46 (31.1)	54 (36.5)	100 (33.8)
Prior psoriasis treatment^b^			
Topical therapy	98 (66.2)	102 (68.9)	200 (67.6)
Light and laser therapy	52 (35.1)	53 (35.8)	105 (35.5)
Nonbiologic systemic therapy	54 (36.5)	60 (40.5)	114 (38.5)
Biologic therapy	32 (21.6)	27 (18.2)	59 (19.9)
Baseline psoriasis severity, mean (95% CI)			
PASI score	4.68 (3.96-5.41)	4.40 (3.80-5.00)	NA
BSA, % affected by psoriasis	9.71 (7.35-12.07)	7.67 (6.14-9.21)	NA
PtGA score	2.18 (2.00-2.35)	2.15 (1.98-2.32)	NA
Insurance type^a^			
Private	77 (52.0)	78 (52.7)	155 (52.4)
Medicaid	28 (18.9)	34 (23.0)	62 (20.9)
Medicare	27 (18.2)	26 (17.6)	53 (17.9)
No insurance	8 (5.4)	5 (3.4)	13 (4.4)
Marital status^a^			
Single	48 (32.4)	56 (37.8)	104 (35.1)
Married	79 (53.4)	69 (46.6)	148 (50.0)
Divorced	10 (6.8)	7 (4.7)	17 (5.7)
Separated	5 (3.4)	5 (3.4)	10 (3.4)
Widowed	0	8 (5.4)	8 (2.7)
Tobacco use^a^			
Never	81 (54.7)	84 (56.8)	165 (55.7)
Former	36 (24.3)	42 (29.1)	78 (26.4)
Current	24 (16.2)	18 (12.2)	42 (14.2)
Chewing tobacco	2 (1.4)	1 (0.7)	3 (1.0)
Educational level^a^			
Grade school	16 (10.8)	13 (8.8)	29 (9.8)
Some high school	3 (2.0)	10 (6.8)	13 (4.4)
High school graduate	23 (15.5)	21 (14.2)	44 (14.9)
College	65 (43.9)	73 (49.3)	138 (46.6)
Graduate school	36 (24.3)	26 (17.6)	62 (20.9)
Working status^a^			
Full time	73 (49.3)	67 (45.3)	140 (47.3)
Part time	26 (17.6)	17 (11.5)	43 (14.5)
Retired	13 (8.8)	18 (12.2)	31 (10.5)
Disabled	10 (6.8)	20 (13.5)	30 (10.1)
Homemaker	8 (5.4)	6 (4.1)	14 (4.7)
Other	8 (5.4)	7 (4.7)	15 (5.1)
Looking for employment	5 (3.4)	10 (6.8)	15 (5.1)
Alcohol use^a^			
Never	36 (24.3)	33 (22.3)	69 (23.3)
Former	38 (25.7)	29 (19.6)	67 (22.6)
Current	69 (46.6)	83 (56.1)	152 (51.4)
Comorbidities^b^			
Heart disease	5 (3.4)	7 (4.7)	12 (4.1)
Arthritis	32 (21.6)	45 (30.4)	77 (26.0)
Internal malignancies	4 (2.7)	8 (5.4)	12 (4.1)
Liver disease	4 (2.7)	8 (5.4)	12 (4.1)
Celiac disease	1 (0.7)	1 (0.7)	2 (0.7)
Stroke	2 (1.4)	3 (2.0)	5 (1.7)
Thyroid problems	12 (9.5)	12 (8.1)	24 (8.1)
Vision problems	22 (14.9)	24 (16.2)	46 (15.5)
Tuberculosis	6 (4.1)	7 (4.7)	13 (4.4)
Inflammatory bowel disease	4 (2.7)	3 (2.0)	7 (2.4)
Basal cell carcinoma	4 (2.7)	5 (3.4)	9 (3.0)
Squamous cell carcinoma	1 (0.7)	2 (1.4)	3 (1.0)
Melanoma	0	2 (1.4)	2 (0.7)

Abbreviations: BSA, body surface area; NA, not applicable; PASI, Psoriasis Area and Severity Index; PtGA, Patient Global Assessment.

^a^ Some participants declined to answer the questions regarding race (1 in the online group; 4 in the in-person group), insurance type (8 in the online group; 5 in the in-person group), marital status (6 in the online group; 3 in the in-person group), tobacco use (3 in the online group; 3 in the in-person group), educational level (5 in the online group; 5 in the in-person group), working status (5 in the online group; 3 in the in-person group), and alcohol use (5 in the online group; 3 in the in-person group).

^b^ Responses are not mutually exclusive.

### Randomization

We enrolled 300 adults with psoriasis. Stratified randomization was performed using computer-generated random block sizes. Patients were randomized 1:1 to collaborative connected health (online intervention) or usual in-person care, stratified by site and psoriasis disease severity (1:1:2 stratification to groups with mild [<3% body surface area (BSA)], moderate [3%-10% BSA], and severe [>10% BSA or receiving phototherapy or systemic therapies] psoriasis). This stratification promotes the recruitment of patients with psoriasis across the disease spectrum and across therapeutic modalities. An independent statistician generated and concealed the randomization sequence and assigned the participants to the interventions.

### Interventions

#### Online Model

The online, collaborative connected-health model was designed such that any specialist services that usually occur in person could be delivered through asynchronous online health care in a flexible and prompt manner. This model was intended to foster expeditious expert advice, multidirectional communication, and sharing of visit information among patients, PCPs, and dermatologists. In this pragmatic trial, the PCPs could access the dermatologists online asynchronously via consultation or requesting a dermatologist to assume care of a patient’s psoriasis. In the consultation setting, similar to traditional asynchronous telemedicine, PCPs or their office staff would send digital photographs and clinical history data to the dermatologists online via a secure, Health Insurance Portability and Accountability Act–compliant web-based connected-health platform.^24^ Within 2 business days, the dermatologist would provide treatment recommendations and patient educational materials online to the PCP and, with the PCP’s permission, to the patients. In settings in which the PCP requested the dermatologist to assume longitudinal care of a patient’s psoriasis, the PCP’s office would send photographs and history data online to the dermatologist, who would then evaluate the transmitted information. The dermatologist would then communicate recommendations, prescribe medications, and provide educational materials online asynchronously to the patient. The dermatologist would also share all visit information with the PCP. Additional follow-up questions with dermatologists were handled online or via telephone.

Patients randomized to the online group had the option of accessing dermatologists online asynchronously. For example, if a patient desired to access a dermatologist, he or she could connect with a dermatologist online with the understanding that the dermatologist would share all visit information and communicate with the patient’s PCP. During an online visit, the patient would upload clinical images and history data and transmit the information to the dermatologist. Using the telehealth platform, the dermatologist would review the transmitted information, make treatment recommendations, prescribe medications, and provide educational materials to patients online asynchronously.

#### In-Person Model (Control Arm)

Patients randomized to the in-person group sought psoriasis care from PCPs or dermatologists in person. The frequency of visits for all the patients in the online and in-person groups was based on medical necessity, as determined by joint decisions between the clinicians (dermatologists or PCPs) and patients. Owing to the nature of the interventions, blinding of patients and clinicians was not possible. Blinding of the data analysts and statistician was preserved for analysis of outcomes.

### Outcomes

All primary and secondary outcomes were defined and prespecified. To compare differences in psoriasis disease severity between the online and in-person arms, the participants completed the Psoriasis Area and Severity Index (PASI), assessed BSA affected by psoriasis, and completed the patient global assessment (PtGA) at quarterly intervals. Studies have shown that standardized training results in an accurate assessment of the PASI score by patients.^25^ At the baseline visit, all participants received training and demonstrated competency in completing these assessments.

The PASI is a validated instrument that combines the assessment of lesion severity (erythema, induration, and scale) and the affected surface area into a single score ranging from 0 (no disease) to 72 (maximal disease). Erythema, induration, and scale are weighted equally in the assessment of plaque morphologic characteristics in a single patient. The prespecified primary outcome of the study was the mean improvement in the PASI score averaged over 3, 6, 9, and 12 months. The improvement in the PASI score was defined as the difference in PASI scores between the baseline visit and each of the follow-up visits. The mean score improvement across the 4 follow-up assessments was selected because it is clinically more meaningful than a single assessment, it would be sensitive to early improvements as well as later benefits, and it is statistically more efficient than a single assessment because the overall patient sample size is generally preserved. We used mixed models for repeated measures as the primary analysis for handling missing data. A 50% change in PASI scores has been suggested as a clinically significant end point in psoriasis.^26^ However, this end point typically pertains to patients in therapeutic trials with moderate to severe psoriasis who are either naive to systemic therapy or have completely discontinued systemic therapies for a minimum washout period (which varies by medication) prior to enrollment. In real-world practice, patients with psoriasis undergo ongoing therapy, and their disease outcomes are currently inconsistently captured.

Prespecified secondary outcomes included BSA affected by psoriasis and the PtGA score. Body surface area is a validated measure used to report the percentage of body surface affected by psoriasis in many prior studies.^27^ Body surface area ranges from 0% (no involvement) to 100% (complete body surface affected). The PtGA is a validated instrument that measures overall psoriasis severity from the patients’ perspective.^28^ The PtGA is an ordinal 6-point scale ranging from 0 (clear) to 5 (severe). Both BSA and the PtGA score were assessed at baseline and at 3, 6, 9, and 12 months.

### Statistical Analysis

For each group, baseline demographic and clinical characteristics were computed and reported as mean values with 95% CIs for continuous outcomes and as numbers and percentages for categorical outcomes. Patterns of adherence to the treatment protocol and missing data were also assessed in both groups. The a priori anticipated attrition rate was 15% across 12 months. The mean unadjusted change from baseline at each time point was computed for the 3 outcomes.

To test the null hypothesis *H*_0_: δ ≤ δ*_L_* or δ ≥ δ*_U_* against the 2-sided alternative hypothesis *H_a_*: δ*_L_* < δ < δ*_U_* at α = .05 for each outcome,^29^ we used an intention-to-treat approach to analyze outcomes using mixed-model repeated-measures analysis (see the study protocol in the Supplement).^30,31,32^ Analyses accounted for site clustering, and the stratifying factor of disease severity was accounted for as an a priori covariate. Diagonal covariance matrices were used, allowing for intrapatient variability to be assessed, while assuming independence across patients. The mixed-effects modeling further enabled us to use all data and not eliminate participants who missed a visit. Visits were coded with baseline as 0 and all postrandomization visits as 1, so that the estimated marginal mean values for time by group would estimate the difference in the mean change from baseline between groups. We computed 95% CIs on the estimated marginal mean values to compare with the a priori equivalence margins, which were derived from clinical trials evaluating systemic medications for psoriasis^33,34,35^ and were considered clinically meaningful by clinicians and patients with psoriasis during pilot work. The equivalence margins were determined a priori to maintain α = .05. For the self-administered PASI score (primary outcome), the equivalence margin was ±6.5. For BSA affected by psoriasis (secondary outcome), the equivalence margin was ±6.5%, and the equivalence margin for the PtGA score (secondary outcome) was ±0.25. These values allowed for us to achieve a power of 75% to 99% depending on σ and ρ, assuming attrition of 15%. All analyses were conducted using SPSS, version 24, software (IBM Corp). All *P* values were from 2-sided tests and results were deemed statistically significant at *P* < .05.

## Results

From February 2, 2015, to August 18, 2017, we conducted participant recruitment and study visits. A total of 300 patients were enrolled (Figure 1), and 296 patients were randomized to either the online (n = 148) or in-person (n = 148) group. All randomized participants received the intended intervention, and all were analyzed for the primary outcome and secondary outcomes. Each participant was followed up for 12 months. The completion rates for the quarterly assessments were excellent among participants, at 274 participants (92.6%) at month 3, 265 participants (89.5%) at month 6, 264 participants (89.2%) at month 9, and 255 participants (86.1%) at month 12. The mean (SD) number of completed assessments was 4.7 (1.0) for the in-person group and 4.5 (1.2) for the online group.

Baseline participant demographic and clinical characteristics for the 2 intervention groups are shown in Table 1. Balance was achieved across these variables between groups; hence, none of these variables were included in post hoc sensitivity analyses of the outcomes. The sample was balanced between men (149 [50.3%]) and women (147 [49.7%]), and the mean (SD) age was 49 (14) years. With regard to race/ethnicity, 187 patients (63.2%) were white, 19 patients (6.4%) were Asian, 8 patients (2.7%) were African American, and 100 patients (33.8%) were Hispanic. A total of 77 patients (26.0%) had arthritis. Stratification based on disease severity was balanced between the 2 groups.

The baseline psoriasis severity scores as measured by the PASI, BSA affected by psoriasis, and the PtGA are summarized in Table 1. Consistent with the pragmatic design, severity of psoriasis was defined based on either BSA involvement or the use of phototherapy or systemic therapy. For example, psoriasis was classified as severe at baseline if the patient had a BSA of more than 10% affected by psoriasis or if the patient was receiving phototherapy or systemic therapy regardless of BSA affected by psoriasis. The therapy-based definition is consistent with most real-world studies of psoriasis; furthermore, patients receiving systemic therapies likely had intrinsic, severe psoriasis. Because many patients had been receiving systemic therapy for some time at enrollment, these patients had low PASI scores. In addition, owing to the pragmatic design, patients were at different stages of their treatment at enrollment. Some patients who had recently started systemic therapies at enrollment had thinner plaques (low morphologic scores on the PASI) but high BSA affected by psoriasis, which elevated the mean BSA affected by psoriasis. Baseline PASI and PtGA scores were consistent with one another. Overall, there were no differences in any psoriasis severity measures between the 2 groups at baseline.

**Figure 3.**
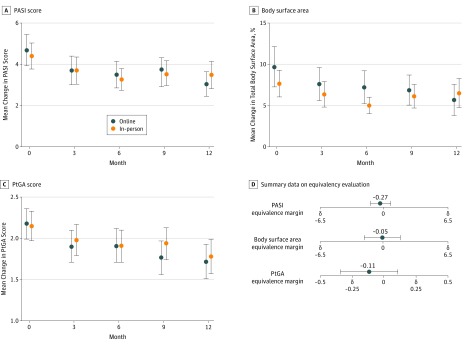
Changes in Psoriasis Disease Severity and Summary Data on Equivalency Evaluation A, Change in psoriasis disease severity as measured by Psoriasis Area and Severity Index (PASI) score by group over 12 months. B, Change in psoriasis disease severity as measured by body surface area by group over 12 months. C, Change in psoriasis disease severity as measured by Patient Global Assessment (PtGA) score by group over 12 months. D, Summary data on equivalency evaluation. Error bars indicate 95% CI.

For the primary outcome in the online group, the mean PASI score changed from 4.68 (95% CI, 3.96-5.41) at baseline to 3.71 (95% CI, 3.05-4.36) at month 3, 3.50 (95% CI, 2.89-4.11) at month 6, 3.62 (95% CI, 2.95-4.29) at month 9, and 3.04 (95% CI, 2.45-3.63) at month 12. In the online group, the mean (SD) change in PASI score from baseline across the follow-up visits was –1.37 (3.33) (Figure 3A). In the in-person group, the mean PASI score changed from 4.40 (95% CI, 3.80-5.00) at baseline to 3.70 (95% CI, 3.08-4.32) at month 3, 3.27 (95% CI, 2.78-3.76) at month 6, 3.58 (95% CI, 3.01-4.14) at month 9, and 3.48 (95% CI, 2.81-4.15) at month 12. In the in-person group, the mean (SD) change in PASI score from baseline across the follow-up visits was −0.82 (3.43). The difference in mean follow-up change in PASI score between the online and in-person groups was –0.27 (95% CI, −0.85 to 0.31), which is within the prespecified equivalence margin (±6.5) (Figure 3A and D). Thus, the online group achieved improvement in psoriasis disease severity equivalent to the in-person group, as measured by the PASI score.

One secondary outcome was the mean change in BSA affected by psoriasis from baseline across 12 months. In the online group, the mean BSA affected by psoriasis changed from 9.71% (95% CI, 7.35%-12.07%) at baseline to 7.61% (95% CI, 5.69%-9.53%) at month 3, 7.23% (95% CI, 5.33%-9.13%) at month 6, 6.88% (95% CI, 5.13%-8.62%) at month 9, and 5.68% (95% CI, 3.88%-7.48%) at month 12. In the online group, the mean (SD) change in BSA affected by psoriasis from baseline across the follow-up visits was –3.38% (11.08%) (Figure 3B). In the in-person group, the mean BSA affected by psoriasis changed from 7.67% (95% CI, 6.14%-9.21%) at baseline to 6.37% (95% CI, 4.90%-7.84%) at month 3, 5.02% (95% CI, 4.10%-5.93%) at month 6, 6.14% (95% CI, 4.80%-7.49%) at month 9, and 6.51% (95% CI, 4.83%-8.20%) at month 12. In the in-person group, the mean (SD) change in BSA affected by psoriasis from baseline across the follow-up visits was −1.55% (8.87%). The difference in mean follow-up change in BSA affected by psoriasis between the online and in-person groups was −0.05% (95% CI, −1.58% to 1.48%), which is within the prespecified equivalence margin (±6.5%) (Figure 3B and D). The online group achieved improvement in psoriasis disease severity equivalent to the in-person group, as measured by BSA affected by psoriasis.

Another secondary outcome was the mean change in PtGA score from baseline across 12 months. In the online group, the mean PtGA score changed from 2.18 (95% CI, 2.00-2.35) at baseline to 1.90 (95% CI, 1.72-2.09) at month 3, 1.91 (95% CI, 1.72-2.11) at month 6, 1.77 (95% CI, 1.57-1.96) at month 9, and 1.72 (95% CI, 1.52-1.92) at month 12. In the online group, the mean (SD) change in PtGA score from baseline across the follow-up visits was −0.37 (1.00) (Figure 3C). In the in-person group, the mean PtGA score changed from 2.15 (95% CI, 1.98-2.32) at baseline to 1.98 (95% CI, 1.80-2.16) at month 3, 1.91 (95% CI, 1.72-2.09) at month 6, 1.94 (95% CI, 1.75-2.12) at month 9, and 1.78 (95% CI, 1.58-1.98) at month 12. In the in-person group, the mean (SD) change in PtGA score from baseline across the follow-up visits was −0.22 (1.26).

The difference in the mean change in PtGA score across 12 months between the online and in-person groups was −0.11 (95% CI, −0.32 to 0.10), which exceeded the prespecified equivalence margin (±0.25) (Figure 3D). Specifically, the online group had greater improvement in psoriasis severity as measured by the PtGA score compared with the in-person group.

During the 12-month study period, the in-person group had a total of 315 in-person visits, while the online group had 161 online visits. Owing to the pragmatic design, the patients in the online group were also permitted to seek in-person care for psoriasis if deemed necessary by the clinician. The online group also had 8 additional in-person visits. Specifically, 3 visits were for in-office procedures, 2 visits were for evaluation of a comorbid condition, 2 visits were for psoriasis exacerbation deemed best managed in person, and 1 visit was for evaluation of a drug-related adverse event.

During the 12-month trial, the types of treatments that the patients received were similar between the 2 groups. The following distribution reflects the most recent treatments that the patients received by the end of the study. Specifically, 64 patients in the online group (43.2%) and 67 patients in the in-person group (45.3%) received topical treatments alone, 7 patients in the online group (4.7%) and 9 patients in the in-person group (6.1%) received phototherapy, 18 patients in the online group (12.2%) and 15 patients in the in-person group (10.1%) received oral therapies, 56 patients in the online group (37.8%) and 53 patients in the in-person group (35.8%) received biologic therapies, and 3 patients in the online group (2.0%) and 4 patients in the in-person group (2.7%) received combination systemic therapies. *Treatment changes* refers to dose changes in an existing medication, the addition of a new medication, or the discontinuation of an existing medication. Online and in-person visits resulted in similar frequencies of treatment changes. In the online group, 78.9% of the online visits (127 of 161) resulted in treatment changes, with dose changes in the same medication being the most frequent; in the in-person group, 81.9% of the visits (258 of 315) resulted in treatment changes, with dose changes in the same medication also being the most frequent. Adverse events and serious adverse events are shown in Table 2. Overall, the rates of adverse events were similar between the online (42 [28.4%]) and in-person (51 [34.5%]) groups.

**Table 2.** Adverse Event and Serious Adverse Event Rates

Adverse Event	Patients, No. (%)		
	Online Group (n = 148)	In-Person Group (n = 148)	Total (N = 296)
Any adverse event	42 (28.4)	51 (34.5)	93 (31.4)
Serious adverse event	13 (8.8)	22 (14.9)	35 (11.8)
Internal malignant neoplasm (excluding skin cancer)	2 (1.4)	2 (1.4)	4 (1.4)
Nonmelanoma skin cancer	1 (0.7)	4 (2.7)	5 (1.7)
Exacerbation of arthritis	5 (3.4)	3 (2.0)	8 (2.7)
Surgery	4 (2.7)	6 (4.1)	10 (3.4)
Death	1 (0.7)	1 (0.7)	2 (0.7)

Additional analyses were conducted to evaluate whether there was effect modification by baseline disease severity. When groups with mild, moderate, or severe psoriasis were examined separately for PASI score and BSA outcomes, all 3 severity groups showed a similar mean magnitude of improvement over time, although there were larger variances for patients with the most severe psoriasis. For PtGA score, the magnitude of improvement and variances were similar across the different severity groups. When further stratification was performed by intervention type, there was no effect modification by disease severity.

## Discussion

In many regions of the world, the inability to consistently access specialty care is among the key reasons that patients experience poor outcomes from their chronic skin diseases.^36,37,38^ Although traditional consultative teledermatology has increased access for some patients, its adoption has been limited. In traditional, consultative, asynchronous teledermatology, patients must locate health care facilities with telemedicine capabilities. There, the medical staff would photograph patients’ skin lesions and send the images and clinical history data to a dermatologist online. The dermatologist would serve as a consultant who provides recommendations to the patients’ PCPs online but generally with no direct patient contact. The PCPs would relay the dermatologists’ recommendations to the patients and implement the treatment plans.

Studies show that key limitations exist with traditional, consultative, asynchronous teledermatology that restrict its scalability.^39^ First, PCPs desire greater support from specialists in the form of having specialists address patients’ concerns directly and promptly. Second, patients are dissatisfied with the lack of direct contact with specialists. Third, the extent and timeliness with which the specialist’s recommendations are relayed to the patients are unknown.

To maximize patient-centeredness and provide robust specialist support, we evaluated an innovative, online, collaborative connected-health model for patients with psoriasis via a pragmatic trial. Psoriasis was selected as a disease model because it is a prevalent, chronic skin disease with substantial morbidity and requires regular care from a specialist.

This trial compared patient-centered outcomes between the online and in-person models using validated disease severity measures. We found that the online and in-person groups achieved equivalent improvement in psoriasis disease severity as measured by the primary outcome of PASI score and a key secondary outcome of BSA affected by psoriasis across 12 months.

The clinical significance of these findings is that the patients in the online group consistently experienced a reduction in psoriasis severity throughout 1 year that is equivalent to that of the in-person group. These findings demonstrated that the collaborative connected-health model is as effective as in-person care in improving patient outcomes. Furthermore, this online model brought specialist care to patients and PCPs in a location-independent, time-independent, and efficient manner.^40^ Because the study population reflects real-world patients who are receiving ongoing therapies, the study findings are particularly applicable to the maintenance of therapy for patients with psoriasis.

The study also found that, compared with the patients in the in-person group, the patients in the online group experienced greater improvement in severity of psoriasis as measured by the PtGA, which comprehensively reflects the patients’ perspective of their disease severity and therefore may account for factors beyond disease signs. Thus, the online model was effective in affecting outcomes important to patients.^41^

There are several considerations in the implementation of this collaborative connected-health model. First, the efficiency of such an online model will depend, at least in part, on the quality of the communication technology. That is, a teledermatology platform that is user friendly and intuitive will enable expeditious online communication. Second, to apply the study findings to other disease areas, implementation and adaptation of the collaborative connected-health model will need to account for cost as well as medicolegal considerations. For example, it is important to adequately account for the clinician’s effort in online care with sound reimbursement policies and safeguard online care with quality checks and medicolegal protection. Third, owing to the dermatology workforce shortage and the variability in individual specialists’ preferences, some dermatologists may not be available or willing to provide direct online care to patients. Fourth, having local telehealth “champions” at clinicians’ offices will help enhance implementation and sustainability of such an online model.

### Limitations

The study findings are to be interpreted in the context of the study design. First, while the overall retention rate for this pragmatic trial is high, the in-person group had a slightly higher retention rate (135 [91.2%]) compared with the online group (131 [88.5%]). However, the analyses were based on intention to treat, and missing data were handled via a priori specified methods for both groups. Second, the baseline PASI scores were lower than anticipated because, owing to the pragmatic design, many patients had been receiving systemic therapies at enrollment, which reflects the real-world cohort of patients with psoriasis seen in US ambulatory clinics. Studies in psoriasis populations with milder disease severity and using available SD data would have yielded PASI score equivalence margins around ±3 or ±4.^42,43^ Even if the equivalence margin for PASI scores had been set at ±1, the improvement in psoriasis severity between the 2 groups seen in this study would still be considered equivalent.

## Conclusions

The online, collaborative connected-health model was equivalent to the in-person model in improving psoriasis clinical outcomes. The online, collaborative connected-health model emphasized patient-centeredness by fostering increased patient engagement and providing comprehensive specialist support. The robust and responsive specialist support for patients and PCPs online was a substantial improvement from some of the existing modalities of specialty health care delivery. Innovative telehealth delivery models that emphasize collaboration, quality, and efficiency can be transformative to improving patient-centered outcomes among those with chronic diseases.

## Notes

Supplement. **Trial Protocol**
