# Supplementary material for: Effectiveness of Online vs In-Person Care for Adults With Psoriasis: A Randomized Clinical Trial
Source: JAMA Netw Open. 2018 Oct 5;1(6):e183062. doi: 10.1001/jamanetworkopen.2018.3062 (PMC6324453; doi:10.1001/jamanetworkopen.2018.3062)
Supplement: Supplement. — Trial Protocol [file jamanetwopen-1-e183062-s001.pdf]

TABLE OF CONTENTS

PAGE
TABLE OF CONTENTS I
LIST OF ABBREVIATIONS III
PROTOCOL SUMMARY IV
1 INTRODUCTION: BACKGROUND INFORMATION AND SCIENTIFIC RATIONALE 7
1.1 Background Information 7
1.2 Rationale 8
1.3 Potential Risks and Benefits 11
1.3.1 Potential Risks 11
1.3.2 Potential Benefits 12
2 OBJECTIVES 14
2.1 Study Objectives 14
2.2 Study Outcome Measures 14
2.2.1 Primary 14
2.2.2 Secondary 15
3 STUDY DESIGN 18
3.1 Collaborative Connected Health (CCH) (Intervention Arm): 19
3.2 In-Person Model (Control Arm): 21
4 STUDY ENROLLMENT AND WITHDRAWAL 22
4.1 Subject Inclusion Criteria 22
4.2 Subject Exclusion Criteria 22
4.3 Strategies for Recruitment and Retention 22
4.4 Treatment Assignment Procedures 25
4.5 Subject Withdrawal 25
5 STUDY SCHEDULE 26
5.1 Informed Consent 26
5.2 Screening Visit (Week 0) 26
5.3 Enrollment/Baseline (Visit 1, Week 0) 26
5.4 Visit 2, Week 12 26
5.5 Visit 3, Week 24 26
5.6 Visit 4, Week 36 26
5.7 Visit 5, Week 48 27
6 ASSESSMENT OF SAFETY 28
6.1 Specification of Safety Parameters 28
6.1.1 Unanticipated Problems 28
6.1.2 Adverse Event 28
6.1.3 Serious Adverse Events 28
6.2 Time Period and Frequency for Event Assessment and Follow-Up 29
6.3 Characteristics of an Adverse Event 29
6.3.1 Relationship to Study Intervention 29
6.3.2 Expectedness of SAEs 29

6.4 Reporting Procedures 29
7 STATISTICAL CONSIDERATIONS 30
7.1 Sample Size Considerations 30
7.2 Statistical Methods 30
7.2.1 Aim 1: Psoriasis Disease Severity Outcomes 30
7.2.2 Aim 2: Dermatology-Specific, Quality-of-Life Outcomes and Mental Health Outcomes 31
7.2.3 Aim 3a: Assess Access-to-Specialist Care and Analyze Access-to-Care Measures 32
7.3 Addressing Missing Data 33
7.3.1 Prevent, Monitor, and Report Missing Data 33
7.3.2 Statistical Methods to Handle Missing Data and Sensitivity Analyses 34
8 PATIENT AND STAKEHOLDER ENGAGEMENT 35
8.1 Patient and Stakeholder Engagement in Monitoring Study Conduct and Progress 35
8.2 Patient and Stakeholder Engagement in the Dissemination of Research Results 35
8.3 Shared Decision-Making and PCORI Engagement Principles 36
9 ETHICS/PROTECTION OF HUMAN SUBJECTS 37
9.1 Ethical Standard 37
9.2 Institutional Review Board 37
9.3 Informed Consent Process 37
9.4 Subject Confidentiality 37
10 DATA HANDLING AND RECORD KEEPING 39
10.1 Data Management Responsibilities 39
10.2 Data Capture Methods 39
10.3 Protocol Deviations 39
11 PUBLICATION/DATA SHARING POLICY 40
12 LITERATURE REFERENCES 41

LIST OF ABBREVIATIONS

AE	Adverse Event/Adverse Experience
BSA	Body Surface Area
CCH	Collaborative Connected Health
CIOMS	Council for International Organizations of Medical Sciences
CRF	Case Report Form
DLQI	Dermatology Life Quality Index
EQ-5D-5L	Euro-QoL, 5 Dimension, 5 Level
HIPAA	Health Insurance Portability and Accountability Act
ICF	Informed Consent Form
IRB	Institutional Review Board
N	Number (typically refers to subjects)
MAR	Missing at Random
MNAR	Missing Not at Random
MMRM	Mixed Models for Repeated Measures
PASI	Psoriasis Area and Severity Index
PCP	Primary Care Provider
PCORI	Patient Centered Outcomes Research Institute
PHI	Protected Health Information
PHQ-9	Patient Health Questionnaire
PI	Principal Investigator
SA-PASI	Self-Administered Psoriasis Area and Severity Index
SAE	Serious Adverse Event/Serious Adverse Experience
SOA	Service Oriented Architecture

PROTOCOL SUMMARY

Title: Improving Specialty-Care Delivery in Chronic Skin Diseases

Précis: We will conduct a pragmatic, randomized controlled trial to evaluate the impact of a collaborative connected health model for psoriasis management compared to in-person care. The pragmatic trial will follow study participants for 12 months and compare psoriasis severity (Aim 1), quality of life (Aim 2), and access to care (Aim 3) between those randomized to the online care model versus in-person care.

Objectives: Primary (Aim 1): Determine whether a collaborative connected health model results in equivalent improvements in psoriasis disease severity compared to in-person care. Psoriasis disease severity will be measured by self-administered psoriasis area and severity index (PASI), body surface area (BSA), and patient global assessment (PGA).

Secondary (Aim 2): Determine whether the collaborative connected health model results in equivalent improvements in quality of life and mental health compared to in-person care. We will compare differences in quality of life by using the dermatology-specific instruments, Skindex-16 and Dermatology Life Quality Index. Furthermore, we will compare differences in depression severity using patient health questionnaire-9 (PHQ-9) between patients randomized to collaborative connected model and in-person care.

Secondary (Aim 3): Determine whether the collaborative connected health model provides better access to care compared to in-person care by comparing differences in access-to-care measures such as transportation and difficulties obtaining specialist care between collaborative connected health model and in-person care. The utility of the collaborative connected health model for increasing specialty-care access from patient, PCP, and dermatologist will be assessed using qualitative analyses.

Population: We will recruit from approximately 380,000 adult psoriasis patients from target populations in Colorado, southern California, and northern California to enroll 300 participants. We plan to evaluate the collaborative connected health model in populations for whom, the effectiveness information regarding the optimal healthcare delivery method is most needed. Thus, in addition to recruiting patients from the general population, we will place a specific emphasis on

	recruiting psoriasis patients living in rural and underserved communities. We will recruit adults with mild, moderate, and severe psoriasis to ensure representation from the entire disease spectrum.
**Number of Sites:**	Three regions: Colorado, southern California, and northern California.
**Description of Intervention:**	The collaborative connected health (CCH) model is designed such that any specialist services that usually occur in person can be delivered through asynchronous online healthcare in a flexible and prompt manner that maximizes patient outcomes and fosters multidirectional communication among patients, PCPs and dermatologists. Study participants randomized to the intervention arm will communicate with their dermatologists online through a secure, web-based telemedicine site called Psoriasis Connect, powered by Direct Dermatology, Inc.
**Study Duration:**	36 months
**Subject Participation Duration:**	12 months
**Estimated Time to Complete Enrollment:**	26 months

Schematic of Study Design:

Figure 2: Overview of Pragmatic Trial Evaluating Effectiveness of Collaborative Connected Health in Psoriasis

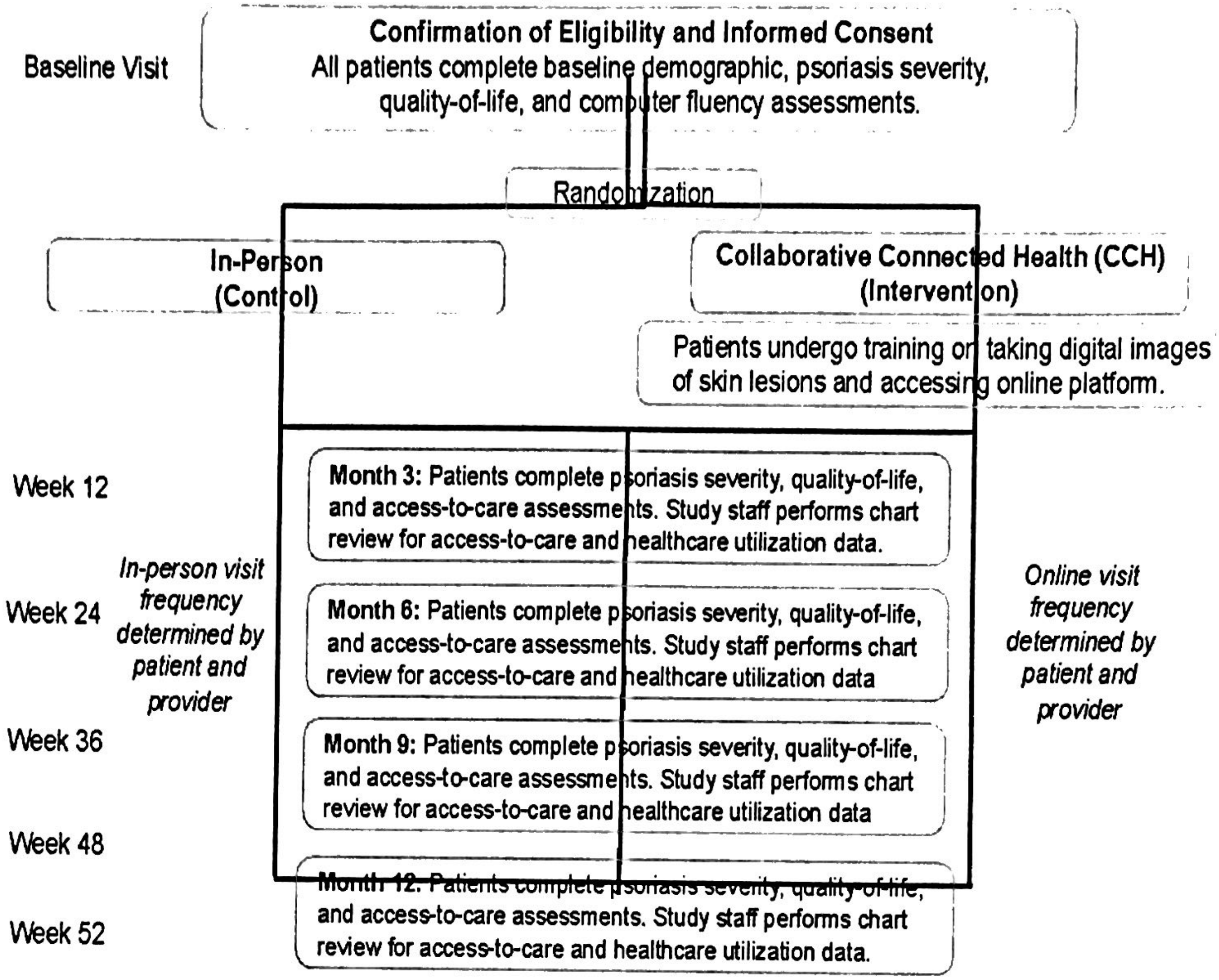

1 INTRODUCTION: BACKGROUND INFORMATION AND SCIENTIFIC RATIONALE

1.1 Background Information

Skin diseases account for 30% of all physician office visits.[2,3] Chronic skin diseases are associated with markedly decreased quality of life and financial consequences.[4] In the U.S., there is lack of access to dermatologists especially among patients living in rural and underserved communities.[5-8] The average wait time for a new patient visit averages 6-8 months in these communities.[7] Even after initial evaluation by dermatologists, patients in remote or underserved areas have difficulties maintaining regular access to dermatologists for follow-up care.[5,8] Consequently, many patients with chronic skin diseases, such as psoriasis, lack regular specialty care and experience worse clinical outcomes and reduced quality of life.[9]

Psoriasis is a chronic, inflammatory skin disease that affects 3-4% of the U.S. population or 10 million Americans.[10] Approximately 42% of psoriasis patients have undiagnosed active disease and do not receive specialist care.[16] Psoriasis manifests as thick, red, scaly plaques that can occur anywhere on the body and are associated with itch, pain, and bleeding (Figure 1). Psoriasis is associated with a number of serious comorbidities including inflammatory arthritis, cardiovascular disease, and severe depression.[12,13] Approximately 30% of psoriasis patients develop psoriatic arthritis, where chronic joint destruction can lead to significant functional disability.[14] Psoriasis is also associated with an increased risk for diabetes, dyslipidemia, hypertension, and metabolic syndrome.[15-17] Psoriasis accounts for an excess of 11,500 annual events of myocardial infarction, stroke, and cardiovascular mortality in the U.S.[17,18] Thus, a team-based approach where dermatologists and PCPs co-manage psoriasis and its comorbidities is critical to improving the overall wellbeing of psoriasis patients.

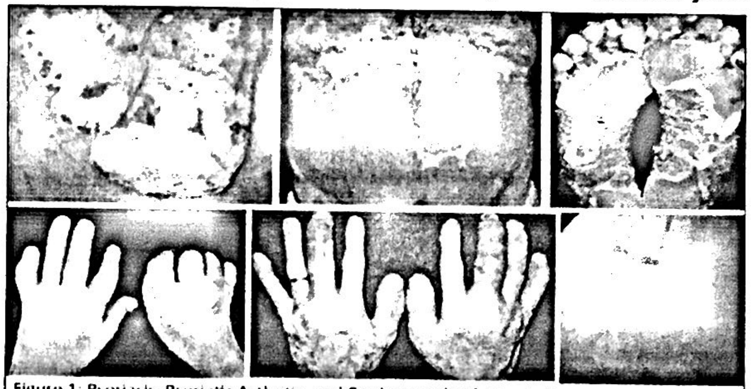

Figure 1: Psoriasis, Psoriatic Arthritis, and Cardiovascular Comorbidities

Connected health is a model for healthcare delivery that uses technology to provide healthcare remotely. Teledermatology is a type of connected health where remote diagnosis and treatment of patients' skin diseases occur by means of telecommunications technology.[19] The application of teledermatology has been met with varied success.[20] While ample evidence supports diagnostic accuracy and reliability of asynchronous teledermatology,[21,22] traditional teledermatology has not been as widely adopted as previously expected.[23,24] Real-world challenges include a lack of collaborative and informed communication among patients, primary care providers (PCPs), and dermatologists in traditional asynchronous teledermatology models.

In traditional asynchronous teledermatology, when a patient presents with a skin condition, the PCP or medical staff takes images of the patient's skin lesions and sends the images and the clinical history to a dermatologist online. The dermatologist serves as a consultant who provides recommendations to the PCP online but has no direct contact with the patient. The PCP then relays the dermatologist's recommendations to the patient and implements the treatment plan. Studies show that several key limitations exist with the traditional asynchronous teledermatology model: (1) PCPs desire greater support from specialists in the form of having specialists address patients' concerns directly and promptly, (2) patients are highly dissatisfied with the lack of direct contact with specialists, and (3) the extent and timeliness with which the specialist's recommendations are relayed to the patients are unknown.[4]

Multiple stakeholders, including patients, their caregivers, and PCPs, have identified collaborative care with specialists as a major goal of improved healthcare delivery.[5,35-38] Furthermore, major professional societies such as the American Academy of Dermatology and the American Telemedicine Association have identified technology-enabled collaborative care for skin diseases as a top priority.[29,39] Thus, given its high prioritization and support by the various stakeholders, a collaborative connected health model is much needed and will likely experience high dissemination success.

1.2 Rationale

In this study, we will evaluate an innovative collaborative connected health (CCH) model where patients and PCPs can access dermatologists online directly and asynchronously via a pragmatic trial. The specific aims are to (1) determine whether the CCH model results in equivalent improvements in psoriasis disease severity compared to in-person care, (2) determine whether CCH results in equivalent improvements in quality of life, and (3) assess whether CCH provides better access to specialists compared to in-person care. These aims contribute directly to the significance of the proposal as detailed below.

1. <u>Improve Specialty-Care and Outcomes via Meaningful Application of Connected Health</u>
To develop more effective methods for specialty-care delivery, it is critical to recognize why current models of telemedicine do not work well in detail. This understanding is essential to appreciate the significance of newer connected health models that aim to improve patient outcomes and address the real-world challenges associated with existing models of teledermatology. Our team evaluated various technology-enabled healthcare delivery methods for providing dermatology services[6,40-53] and their impact on chronic skin diseases.[1,2,6,15,17,20-22,24,28,44,45,54-106] We found that half of the new teledermatology programs discontinued within 5 years, and that most remaining programs continued to face challenges in sustainability.[36] We explored how wide variations in practice patterns suggest clinical uncertainty and why traditional asynchronous teledermatology did not work well from the perspectives of PCPs, patients, and dermatologists.[6,35,37]

First, patients desire easier access to specialists through connected health. To access dermatologists via traditional asynchronous teledermatology, patients need to first find a healthcare facility with telemedicine capabilities. Thus, many patients in rural or underserved communities are limited by the unavailability of telemedicine services in their PCP's offices. Furthermore, even if patients are able to participate in traditional asynchronous teledermatology through their PCP, there is usually no contact between patients and the specialists. Patients learn about the specialists' recommendations from their PCPs, but the patients themselves have

no formalized opportunity to ask follow-up questions directly to a dermatologist. Studies have shown that patients and their caregivers are most troubled by this lack of communication with the specialist, and that this lack of contact is the key source for their dissatisfaction with traditional asynchronous teledermatology.[4] Patients and their caregivers desired easier access to dermatologists online.[4,5] In one study, we found that patients and their caregivers rated having direct access to dermatologists online as "most important" (highest rating on a visual analog scale) for their overall satisfaction with healthcare delivery.[6]

Second, primary care providers seek greater support for team care via connected health. PCPs reported that, while traditional consultative telemedicine has brought greater access to specialty care, they face greater workload challenges conveying and implementing specialists' recommendations from multiple telemedicine-enabled specialties. Frequently, patients have follow-up questions regarding the specialist-originated recommendations that PCPs are not able to answer readily without consulting the specialist again. Thus, without increased support from dermatologists, many PCPs are unable to sustain their efforts in the traditional telemedicine model. The PCPs expressed that they would like a team-care model where dermatologists could become more engaged in communicating with both PCPs and patients. Specifically, PCPs desire dermatologists' support in explaining the diagnoses to patients, implementing the recommendations, and answering patients' follow-up questions.[30]

Third, dermatologists desire to know more about patient progress through connected health. We found that dermatologists noted several key barriers to optimal care delivery using traditional asynchronous consultative teledermatology.[37] Unless the dermatologists share the same medical record system as the referring providers, the dermatologists usually do not know how their recommendations were implemented, whether the patients received adequate education on their disease or treatment regimen, and how patients responded to treatment. For dermatologists, knowing patient progress in response to their recommendations is essential, especially for those with chronic skin diseases where optimal outcome depends on adjusting treatments based on patient response. The dermatologists also reported that poor image quality negatively affected their evaluation. Thus, the ability to communicate with PCPs and patients online to obtain information on patient progress and implementation of standardized image training are paramount to dermatologists.[37]

While traditional asynchronous consultative teledermatology can be helpful, a new delivery model is needed to improve patient outcomes via meaningful application of connected health and address real world challenges. In this application, we will evaluate a collaborative connected health model where online access to dermatologists is direct, versatile, and expedient. To be responsive to real-world workflow, the connected health model offers several ways that PCPs and patients can access dermatologists directly. Specifically, PCPs can access dermatologists online asynchronously for consultations or to request a dermatologist to assume care of patient's psoriasis. Patients can upload clinical images and history online and obtain asynchronous evaluation and recommendations from dermatologists directly. Importantly, the model encourages active sharing of management plans and fosters multi-directional, informed communication among patients, PCPs, and dermatologists to increase engagement from all stakeholders.

Because psoriasis is a common and serious chronic disease that affects patients from diverse socioeconomic and ethnic backgrounds, it serves as an ideal disease to investigate how innovative healthcare models can be used to care for many types of chronic diseases. The distance-independent, time-independent, collaborative connected health model could transform how patients and PCPs interact with dermatologists by providing meaningful technology to

support accessible, collaborative, and patient-centered care. Furthermore, studies have shown that favorable health outcomes are often dependent on active engagement by patients and their caregivers.[107] The collaborative connected health model encourages active engagement by patients, PCPs, and dermatologists by facilitating multi-directional, informed communication.

When the aims are successfully achieved, this connected health model could be applied to other chronic conditions where regular access to specialists is critical to patient outcomes. For example, atopic dermatitis is a chronic condition that affects 30 million Americans.[108-111] Patients and their caretakers take time away from work and school regularly to see dermatologists for follow-up management. The collaborative connected health model not only saves patients time by enabling them to obtain online access to dermatologists from their homes; it also actively engages patients, their caregivers, and PCPs in skin care. Another population particularly suitable for collaborative connected health includes patients with chronic wounds such as diabetic foot ulcers and venous leg ulcers who cannot travel easily. Being engaged in one's own wound care management and being supported via online expertise are likely to improve clinical outcomes and reduce long-term complications. Thus, successful completion of the study aims will provide effective solutions in specialty services delivery and improve outcomes for patients with many types of chronic diseases.

2. <u>Maximize Patient-Centeredness through Collaborative Connected Health Model</u>
The collaborative connected health model maximizes patient-centeredness as compared to both the usual in-person model as well as traditional asynchronous teledermatology (Figure 2). In the usual in-person healthcare setting, patients need to travel to healthcare facilities to receive medical care. They may experience difficulties accessing specialists due to geographic, physical, financial, or scheduling constraints. Furthermore, the quality of communication exchange between PCPs and dermatologists is often inconsistent. Traditional asynchronous teledermatology attempts to address healthcare access by offering consultative dermatology services through communication technology. In traditional asynchronous teledermatology, the dermatologist acts as a consultant to the PCPs online, but they have no direct contact with the patient. While this model has resulted in greater access in some communities, its adoption has met with significant challenges due to factors such as a lack of communication between patients and dermatologists and a lack of sufficient dermatologist support for PCPs.

The goal of the collaborative connected health model is to make any dermatologist interactions that typically occur in-person easily accessible online. This technology-enabled healthcare-delivery model brings specialist care to patients and PCPs in a location-independent and time-

Figure 2: Comparison of in-person, traditional asynchronous teledermatology, and collaborative connected health models

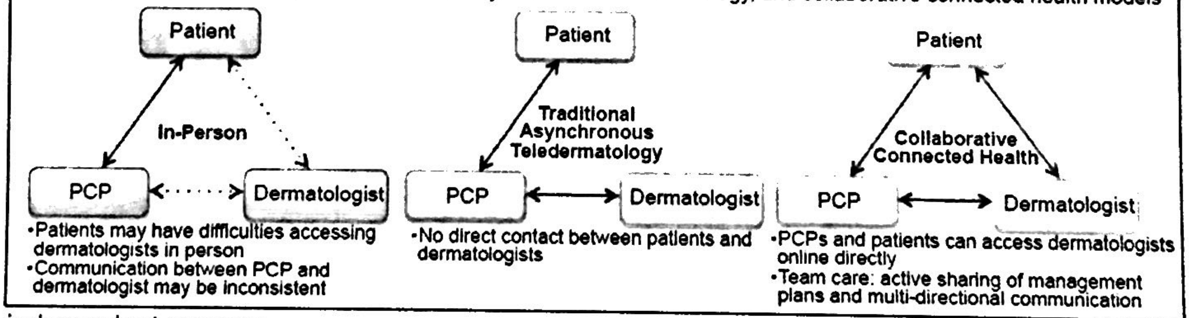

independent manner.

The model's patient-centeredness is innovative in two major aspects. First, the model offers multiple ways for both patients and PCPs to access dermatologists online asynchronously that are responsive to real world needs. PCPs can upload patient's photos and history online and access dermatologists asynchronously for consultations or to request a dermatologist to assume care of a patient's psoriasis. Importantly, patients can also upload images and history online and obtain asynchronous recommendations from the dermatologists. Second, the collaborative connected health model is based on team care, where active sharing of management plans and multidirectional, informed communication among patients, PCP, and dermatologists are a defining feature. For each online visit, the team-care approach requires the dermatologist to share the recommendations and management plans with both the PCP and patient to ensure that all parties are engaged and informed. Both the patient and PCP can also ask follow-up questions either asynchronously online or through telephone. The PCP and the patient will also have access to all online visit records.

While evidence is mature regarding the diagnostic accuracy and reliability of teledermatology, little is known regarding health outcomes associated with teledermatology.[52,112-115] This proposal underscores patient centeredness through the use of patient-centered, validated outcome instruments to compare disease severity, quality of life, mental health, and access to care between collaborative connected health and in-person models. Overall, the collaborative connected health model emphasizes patient-centeredness through fostering increased patient engagement and providing comprehensive specialist support. The robust and responsive dermatologist support for patients and PCPs online is a significant improvement from existing types of dermatology health-services delivery. This collaborative connected health model also eliminates the need for patients to find a local healthcare facility with telemedicine capabilities in order to engage in telemedicine. We will evaluate this model via a pragmatic trial approach that maximizes applicability and generalizability.

1.3 Potential Risks and Benefits

1.3.1 Potential Risks

As with all electronic health information exchange and record platforms, potential risks include security breaches and loss of confidentiality. However, our research team is committed to protecting privacy and maintaining confidentiality of our patients' personal information, and we have applied the highest security standards during technology evaluation to select the following teledermatology platform to use in our CCH model. The secure, HIPAA compliant, online platform that we will be used for this pragmatic trial is called Psoriasis Connect *(*www.psoriasisconnect.org*)*, powered by Direct Dermatology. We have applied the most stringent and updated security assessment parameters in our evaluation process, and Psoriasis Connect's telemedicine platform meets all of the following security parameters (Table 3).

Table 3: Security Features of Direct Dermatology Teledermatology Platform used in the Collaborative Connected Health Arm

Parameter	Security Features of Direct Dermatology Teledermatology Platform
Encryption	Communication between user and connectivity server is encrypted using Advanced Encryption Standard (AES).
Data Security	Direct Dermatology secures data on its servers and in the data center with the following measures: data center is physically and electronically secured. The servers are isolated from the Internet by using a firewall, which blocks access by unauthorized parties.
Confidentiality	Direct Dermatology maintains and enforces explicit policies that keep patients' data and communication private and confidential, and not share the data with any other party.
Digital Certificates	Direct Dermatology uses a digital certificate issued by a Secure Server Certification Authority. This gives the users the confidence that they are connected to a site operated by Direct Dermatology, and authenticated as such.
Login ID and Password	Access to the user account is controlled by a login ID and a password. Strict login ID and password rules are in place to prevent unauthorized user from gaining access.
Auto-Logoff	Direct Dermatology protects the users against accidentally leaving personal information active on a computer browser screen. Direct Dermatology ends a user's "session" if the user is logged in to the site but has not actively used the service for 10 minutes. This prevents others from accessing the account if the user leaves a session and forgets to log out.
Audit Trail	Direct Dermatology maintains an audit trail and log of accesses to a patient's medical profile.
Disaster Recovery Plan	Direct Dermatology is equipped with high levels of disaster recovery, which ensures uptime by preventing outages caused by power, security, environment, fire, and natural disasters. Direct Dermatology delivers the highest levels of reliability through redundant power on the premises and multiple backup generators.

Another potential risk is that digital photographs submitted by the patients may be of poor quality and inadequate for clinical decision-making. To safeguard against poor-quality images, we will hold a standardized photography training session for the online patients at the baseline visit. Training material will be available for review online at any time. Because this is a pragmatic trial, any images that are unsuitable for clinical assessment will require re-imaging by patients. In such cases, the dermatologists will ask the patients to retake the images and resubmit the information for assessment.

1.3.2 Potential Benefits

In the U.S., there is a lack of access to specialty-care providers, especially for patients residing in rural and/or underserved areas. Long wait times are common, and long travel distances renders medical dermatologist visits challenging and cost-prohibitive. To address this critical lack of specialty-care availability, the online access model we're testing offers a number of benefits. First, the collaborative connected health model offers multiple ways for both patients and PCPs to access dermatologists online asynchronously that are responsive to real-world needs. PCPs can upload patient's photos and history online and access dermatologists asynchronously for consultations or to request that a dermatologist assumes care of a patient's psoriasis. Importantly, patients can also upload clinical images and history online and obtain asynchronous evaluation and recommendations from the online dermatologists. Therefore, consultations or requests for a dermatologist to assume care of a patient's psoriasis can be submitted in a timely, cost effective manner.

Second, the collaborative connected health model is based on team care where active sharing of management plans and multidirectional, informed communication among patients,

PCP, and dermatologist are a defining feature. Both the patient and the primary care provider can ask questions either asynchronously or through telephone at any time to the dermatologist. Providers and patients will also have access to all online visit records and management plans to ensure that all parties remain engaged and informed in the patient's care.

Overall, the CCH model not only increases patient and provider engagement; it also provides comprehensive specialist support to both PCPs and patients. The responsive and robust dermatologist support for PCPs and patients online is a significant improvement from existing types of dermatology health-services delivery. With this model, patients can have greater access to dermatologists online for long-term management of chronic skin diseases. After careful consideration of the benefits and risks associated with the study, we deem the benefit-risk assessment to be acceptable to research participants. The accessibility of quality and timely specialist care for psoriasis patients outweighs the potential adverse risks associated with the study.

2 OBJECTIVES

2.1 Study Objectives

The primary goal of the proposed research is to test whether a collaborative connected health model results in equivalent improvements in disease severity, quality of life, and mental health, and whether the model provides better access to specialty care, compared to usual in-person care for psoriasis management via a pragmatic, randomized controlled trial.

Aim 1: Determine whether a collaborative connected health model results in equivalent improvements in psoriasis disease severity compared to in-person care. We intend to compare differences in psoriasis disease severity, as measured by self-administered psoriasis area and severity index (PASI), body surface area (BSA), and patient global assessment (PGA), between the collaborative connected health model and in-person care in a 12-month pragmatic randomized controlled trial.

Aim 2: Determine whether the collaborative connected health model results in equivalent improvements in quality of life and mental health compared to in-person care. Differences in quality of life will be compared using dermatology-specific instruments (Skindex-16 and Dermatology Life Quality Index) between patients randomized to collaborative connected health and in-person care. Additionally, differences in depression severity will be compared using patient health questionnaire-9 (PHQ-9) between patients randomized to collaborative connected health and in-person care.

Aim 3: Determine whether the collaborative connected health model provides better access to care compared to in-person care. Access-to-care measures such as transportation time and difficulties obtaining specialist care will be compared between collaborative connected health model and in-person care. Furthermore, we will also assess utility of connected health model, Psoriasis Connect, for increasing specialty-care access from patient, PCP, and dermatologist perspectives using qualitative analyses.

2.2 Study Outcome Measures

2.2.1 Primary

The primary aim of the study is to determine differences in psoriasis disease severity as measured by self-administered psoriasis severity and area index, body surface area, and patient global assessment between collaborative connected health and in-person care through a pragmatic randomized controlled equivalency trial. We chose an equivalency trial design based on data from our group's preliminary studies.[1,26]

To assess psoriasis disease severity, we will ask patients to complete self-administered Psoriasis Area and Severity Index (SA-PASI), body surface area (BSA) involvement, and patient global assessment (PGA). All patients will receive training on how to complete these instruments at baseline. SA-PASI is a validated instrument that enables psoriasis patients to assess psoriasis disease severity themselves.[30,31] As shown by previous validation studies and our pilot study, patient self-administered PASI correlates well with investigator-assessed PASI and has been used reliably to determine psoriasis severity from patients' perspective.[25,30,31] SA-PASI combines the assessment of lesion severity (erythema, induration, and scale) and the affected areas into a single score between 0 (no disease) to 72 (maximal disease).[30] The primary outcome of the study is the mean percent improvement in SA-PASI averaged over 3, 6,

9, and 12 months. The percent improvement in SA-PASI is defined as the difference in SA-PASI scores between the baseline and each of the follow-up visits divided by the SA-PASI score from the baseline visit. We chose the mean across the four follow-up assessments for two reasons: (1) it would be sensitive to early improvements as well as later benefits, and (2) it is statistically more efficient than an endpoint based on a single assessment.

The body surface area (BSA) assessment is a well-established, validated measure used by psoriasis patients to report percent body surface affected by psoriasis in numerous prior studies.[32] BSA ranges from 0% (no involvement) to 100% (complete body surface affected). The patient global assessment (PGA) is a validated instrument that measures the overall psoriasis severity from the patients' perspective.[33] PGA is an ordinal 6-point scale ranging from 0 (clear) to 5 (severe).

2.2.2 *Secondary*

2.2.2.1 <u>Determine whether the collaborative connected health model results in equivalent improvements in quality of life and mental health compared to in-person care.</u>

Quality-of-life assessments are critical in the evaluation of novel, technology-enabled healthcare delivery models. We have obtained permissions to use dermatology-specific quality-of-life instruments Skindex-16 and Dermatology Life Quality Index (DLQI) for this proposed study.[35] Skindex-16 is a validated and reliable instrument that comprehensively captures the effects of skin disease on health-related quality of life.[36] It discriminates among patients with different effects and is responsive to clinical changes over time.[37] Skindex-16 scores range from 0 (no effect) to 100 (effect experienced all the time), and the responses are aggregated in symptoms, emotions, and functioning scales. We will also use DLQI, another validated dermatology-specific quality-of-life instrument that has been used in many psoriasis trials. DLQI scores range from 0 to 30, with higher scores indicating more severe impact on quality of life.[11] Because the psychometric properties of the two instruments differ in some aspects, using both instruments enables comparison of study findings with previous work in dermatology.[38]

The Patient Health Questionnaire (PHQ) is a validated, self-administered version of PRIME-MD diagnostic instrument for common mental disorders.[39] The PHQ-9 is the depression module, which scores each of the 0 DSM-IV criteria as "0" (not at all) to "3" (nearly every day). A PHQ-9 score of 10 or greater has 89% sensitivity and 88% specificity for major depression. PHQ-9 scores of 5, 10, 15, and 20 represented mild, moderate, moderately severe, and severe depression, respectively. PHQ-9 is a validated tool for diagnosis of depression and monitoring response to interventions.

2.2.2.2 <u>Determine whether the collaborative connected health model provides better access to care compared to in-person care.</u>

To determine whether the CCH provides better access to specialist care compared to the in-person model, we will ask patients to complete access-to-care questions derived from the Medical Expenditure Panel Survey (MEPS) and Medicare Current Beneficiary Survey (MCBS) once every 3 months.[40]

Specifically, we will use the following access-to-specialist-care measures: wait time and transportation factors and difficulties associated with obtaining specialist care.[41,42] The wait time and transportation factors will be evaluated along the following three dimensions:[140] (a) total distance travelled to see a dermatologist as defined by the round-trip distance from a patient's

home to the dermatologist's office multiplied by the number of in-person visits during the 12-month period, (b) total transportation time as defined by the round-trip transit time multiplied by the number of in-person visits to a dermatologist's office, and (c) transportation mode as categorized into walking, driving using one's own car, or taking taxi or public transportation.

While transportation factors pertain mostly to patients in the in-person arm, some patients in the CCH model may require in-person specialist evaluation during the study. Difficulties associated with obtaining specialist care will be assessed based on questions and scales derived from the MEPS and MCBS. We will ask patients the following questions: "How difficult is it to see a dermatologist in-person/online initially?" "How difficult is it to contact the dermatologist about a skin problem?" "How difficult is it to get an online/in-person appointment with a dermatologist on short notice?" The response choices derived from MEPS are on an ordinal scale.[42]

2.2.2.3 Assess the utility of the connected health model for increasing specialty-care access from patient, primary care provider, and dermatologist perspectives using qualitative analyses.

We will perform qualitative data collection and analysis to obtain patient and clinician perspectives on the utility of CCH for increasing specialty-care access. Specifically, we will conduct semi-structured interviews using an interview guide that allows for flexibility in exploring the emerging themes. The interviewer will take field notes on a standard assessment sheet. The interviews will also be audio- recorded for later review, clarification, and analysis. A brief case summary incorporating key findings will be completed within 24 hours of the interview. This will be reviewed by the PI to identify any areas of disagreement or uncertainty about the interpretation of findings. The interviews will be conducted at 6 months and then repeated at 12 months with the same participants.

Patient perspective: We will interview psoriasis patients with varying disease severity as well as varying computer fluency (nine with computer fluency scores above the median and nine with scores below the median). We will seek patient feedback regarding (1) utility of CCH for accessing dermatologic care, (2) user experience with online platform including ease of completing online visits, and (3) perceived quality of healthcare via CCH.

Primary Care Provider and Dermatologist Perspectives: We will interview PCPs and dermatologists to seek their perspectives on the utility of CCH for increasing access to specialty-care. We will seek feedback from these clinicians on (1) utility of CCH for accessing dermatologists for consultations, (2) utility of CCH for providing care online to patients with chronic skin diseases, (3) assessment of clinician effort using CCH, and (4) integration of CCH into existing workflow.

We will employ qualitative analytical techniques with investigator triangulation and member checking to enhance the validity of the conclusions drawn. Specifically, once the data have been collected from the various stakeholders, a template style of analysis will be used to organize the data for reflection and development of emerging themes.[43,44] The initial list of codes will be based on broad thematic areas we expect to see in the data based on our

questions. Template coding also allows for using additional codes for emerging themes analysts observe early in the coding. Coded data will be further reviewed to refine and develop provisional themes for further reflection.

3 STUDY DESIGN

This is a 12 month, pragmatic, randomized controlled, equivalency trial evaluating the impact of a collaborative connected health model for psoriasis management compared to in-person care. The pragmatic trial will compare psoriasis severity (Aim 1), quality-of-life and mental health (Aim 2), and access-to-care (Aim 3) between the two models. The rationale for proposing a pragmatic trial is to test whether this collaborative connected health model works in real life. This design allows for a large spectrum of everyday clinical settings in order to maximize applicability and generalizability. The pragmatic approaches are especially pronounced along these PRECIS domains:[27] the inclusive eligibility criteria, experimental intervention flexibility, usual in-person care as the control intervention, and primary outcome being highly relevant to patients. We describe the pragmatic trial in accordance with the CONSORT statement.[28]

We will recruit from approximately 380,000 adult psoriasis patients from target populations in Colorado, southern California, and northern California to enroll 300 participants in the study. In addition to recruiting from the general population, we will place a specific emphasis on recruiting patients living in rural and underserved communities; we will also recruit from the full psoriasis disease spectrum.

Patient recruitment and enrollment will begin the fourth month of year 1 and continue through the sixth month of year 2. The expected duration of this pragmatic, randomized controlled equivalency trial for each participant will be 12 months, including an initial baseline visit and follow-up visits at 3, 6, 9, and 12 months. No additional follow-up visits beyond the treatment duration are expected for this research study.

During the baseline visit, month 3, 6, 9 and 12, patient reported outcomes will be collected. Psoriasis severity will be assessed using the self-administered Psoriasis Area and Severity Index and the Body Surface Area (BSA) evaluation, both widely accepted measurement tools for psoriasis. Other patient-reported outcomes including the EuroQol EQ-5D-5L, the Dermatology Life Quality Index (DLQI), the Skindex-16, the Patient Health Questionnaire (PHQ-9) and access-to-care questionnaires will be assessed at baseline and months 3, 6, 9 and 12. Data collection will occur primarily through an online database called Research Electronic Data Capture, a secure, web-based application designed to support data capture for IRB-approved research studies. The data collection schedule for all participants in this 12-month pragmatic trial is summarized in Table 1.

Table 1: Data Collection	Baseline	3 Month	6 Month	9 Month	12 Month
Demographic, Socioeconomic, Computer-Email-Web Fluency	X				
Psoriasis severity: SA-PASI, BSA, PGA	X	X	X	X	X
Quality of life: Skindex-16, DLQI, EQ-5D-5L	X	X	X	X	X
Mental Health (Depression): PHQ-9	X	X	X	X	X
Access to care: wait time and transportation, obtaining care	X	X	X	X	X

Qualitative assessment of utility of CCH			X		X

3.1 Collaborative Connected Health (CCH) (Intervention Arm):

The collaborative connected health (CCH) model is designed such that any specialist services that usually occur in person can be delivered through asynchronous online healthcare in a flexible and prompt manner that maximizes patient outcomes and fosters multidirectional communication among patients, PCPs, and dermatologists. CCH enables patients and PCPs to receive prompt dermatologist expertise online, and the dermatologist shares all online visit information with both the patient and PCP. To be responsive to real-world clinical practice, the CCH model offers three modalities by which PCPs and patients can access dermatologists online.

Patients randomized to CCH will first undergo training on how to 1) take standardized digital images of skin lesions, and (2) communicate with their dermatologists online through a secure, web-based telemedicine site, Psoriasis Connect.[24] All training materials are sent to the patients electronically and in hardcopies; they are accessible via the online platform at any time. During the training, patients and their designated family members will be taught how to take at least 8 digital skin photographs for each visit: 4 "global" images (front, back, left, and right sides of the body) and close-up images of representative lesions from each body region: (1) head and neck, (2) upper extremities, (3) lower extremities, and (4) chest, abdomen, and back (Figure 4). The global images are used to assess the body surface area for psoriasis involvement, whereas the close-up images are used for morphologic examination. Of note, the patient's PCP and their designated staff will have full access to the online platform, where they can view all visit information. In addition, the PCPs and their staff will also have access to the standardized training materials at any time through the telemedicine site.

Figure 4: Image Sets for Online Visits in the Collaborative Connected Health (CCH) Model

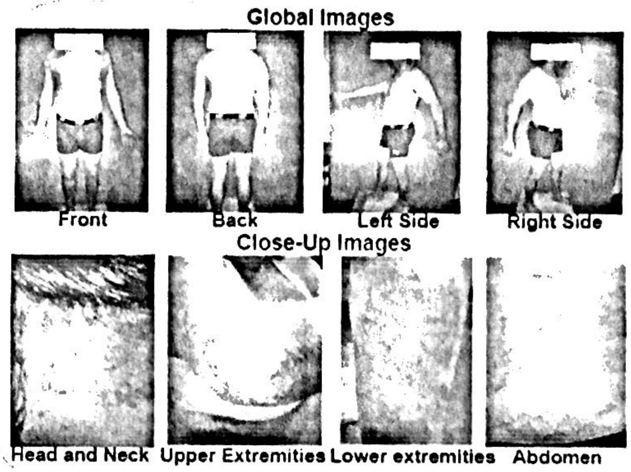

In this pragmatic trial, three ways exist to increase PCP and patient access to dermatologists online (Figures 5 and 6). For PCPs whose psoriasis patients are randomized to CCH, the PCPs can access the dermatologists online asynchronously and promptly in two ways—(1) consultation or (2) request for evaluation and transfer-of-care.

PCP-Initiated Dermatology Consultations in CCH: In the consultation setting, similar to traditional asynchronous telemedicine, a PCP requests online dermatology consultation for a specific clinical scenario (such as during psoriasis exacerbation), but the PCP still wishes to maintain primary care of the patient's psoriasis. The PCP or the office staff sends digital photos and clinical history to the dermatologist online via a secure, HIPAA-compliant web-based connected health platform. Instructions on how to take the photos and transmit the information are available on the website. Within two business days, the dermatologist provides the treatment recommendations and patient educational materials online to the PCP and, with PCPs' permission, to the patient.

Figure 5: Multidirectional communication in the Collaborative Connected Health Model

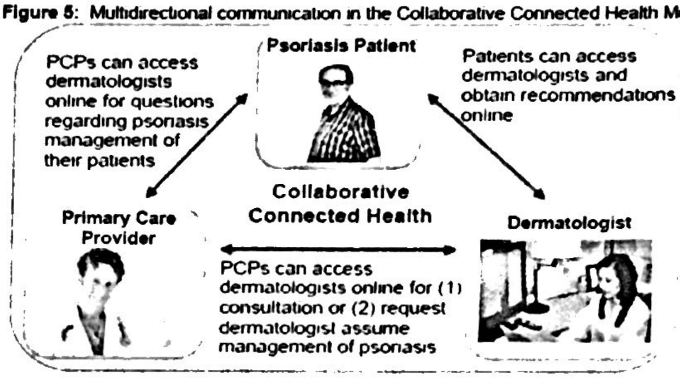

PCP-Initiated Request for Dermatologist Management in CCH: A PCP may wish to have a dermatologist assume care for a patient's psoriasis. In this setting, the PCP requests a dermatologist to assume longitudinal care of a patient's psoriasis. The PCP's office sends photos and history online to the dermatologist, who evaluates the transmitted information. If the dermatologist has not interacted with the patient previously, the dermatologist will call the patient to establish a relationship and clarify any concerns. The dermatologist will then communicate recommendations online asynchronously, e-prescribe medications, and provide educational materials online. Finally, the dermatologist will send all visit information to the PCP and the patient. If the patient or PCP has follow-up questions, they can communicate with the dermatologist online or via telephone.

Patient-Initiated Request for Dermatologist Online Care in CCH: Patients randomized to CCH have the option of accessing dermatologists online asynchronously. For example, if a patient desires to access a dermatologist, he or she can connect with a dermatologist online with the understanding that the dermatologist will share all visit information and communicate with the PCP. If a patient randomized to CCH is already under the care of a dermatologist, the patient's regular dermatologist will be invited to care for the patient online and be provided with training on performing teledermatology. However, if the regular dermatologist declines to engage in CCH, the study dermatologist will work with the regular dermatologist to ensure adequate care transition to enable online management of the patient's psoriasis by the study dermatologist.

Figure 6: Asynchronous online visits in the Collaborative Connected Health (CCH) Model

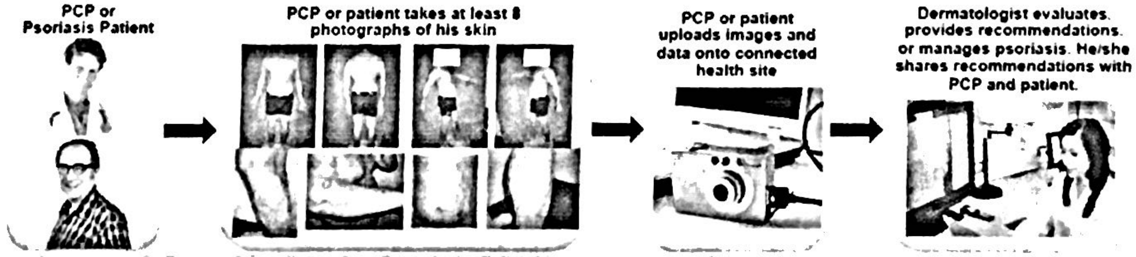

During an online visit, the patient uploads clinical images and history and transmits the information to the dermatologist. Within two business days, the dermatologist will review the

patient's history and images. The dermatologist then communicates recommendations to the patient, prescribe medications, and provide educational materials online asynchronously. The frequency of online visits will be determined between the patient and the dermatologist. If the patient has questions regarding the recommendations or medications, he or she can contact the dermatologist through secure messaging on Psoriasis Connect, or by telephone. At the end of each online visit, the dermatologist sends all visit information to the patient's PCP and is also available to answer PCP questions online or by telephone.

Importantly, from our pilot study, we anticipate that in this pragmatic trial, some patients randomized to the online group will occasionally see a provider in-person for his/her psoriasis care. This will usually occur in the context of an unexpected or severe psoriasis flare or for the evaluation of a skin concern unrelated to psoriasis. We expect that these in-person visits will occur uncommonly. We have an access-to-care questionnaire that proactively captures information from patients regarding whether in-person visits have been conducted in conjunction with using the online CCH platform. If a participant from the CCH arm notes an in-person psoriasis visit, the research team will obtain a release of medical information from the participant and contact the appropriate office to gather the medical record from that visit. These occasional in-person visits will be recorded and analyzed. However, in-person visits will not affect the patients' participation in the study.

3.2 In-Person Model (Control Arm):

Consistent with the pragmatic design, patients randomized to the in-person group will continue to seek psoriasis care as usual from either a primary care provider or from a dermatologist. The providers and patients determine the frequency of the in-person visits, just as they would occur in the real world. In this study, blinding of patients and providers is not possible due to the nature of the intervention.

4 STUDY ENROLLMENT AND WITHDRAWAL

4.1 Subject Inclusion Criteria

In order to be eligible to participate in this study, an individual must meet all of the following criteria:

- Provide signed and dated informed consent form
- Male or female, at least 18 years of age
- Have active, physician-diagnosed plaque psoriasis
- Have access to the internet
- Have access to a digital camera or a phone with camera capabilities
- Have a primary care physician (PCP) or ability to establish care with a PCP

4.2 Subject Exclusion Criteria

An individual who meets any of the following criteria will be excluded from participation in this study:

- He/she does not have active plaque psoriasis at the beginning of the study
- Does not live in Colorado or California
- Unable to fulfill the required tasks of the study

4.3 Strategies for Recruitment and Retention

We plan to enroll 300 participants in the study from a target population of approximately 380,000 adult psoriasis patients in Colorado, southern California, and northern California. In addition to recruiting patients from the general population, we will place an emphasis on recruiting psoriasis patients living in underserved and rural communities because these are the populations for whom effectiveness information regarding the optimal healthcare delivery method is most needed. Two key strengths of the study population are that the patients will (1) be diverse in demographic and socioeconomic factors and (2) represent the full spectrum of psoriasis disease severity. To achieve this, we will focus on recruiting from federally qualified health centers, clinics serving rural communities, free clinics serving the indigent population, in addition to recruiting from general dermatology clinics. We will also perform stratified randomization of patients with mild, moderate, and severe psoriasis. Finally, we recognize that participation may be biased by computer literacy and the possession of a camera and a computer for personal use. We will characterize those who do not agree to participate or who do not qualify for the study so that this potential bias can be analyzed and reported.

Recruiting from Colorado Psoriasis Patients:

To engage psoriasis patients from Colorado, we are partnering with the State Network of Colorado Ambulatory Practices and Partners (SNOCAP), University of Colorado clinics, and Denver Health. SNOCAP is an affiliation of Practice Based Research Networks (PBRNs) in Colorado. SNOCAP members include the High Plains Research Network and the Colorado Research Network. Specifically, the High Plains Research network is a PBRN that covers a 15,000 square-mile eastern region of Colorado. It consists of a network of 200 PCPs from 55 rural primary care practices and 16 hospital systems caring for a rural population of 100,000

people from 16 counties in rural and frontier Colorado. The Colorado Research Network is a PBRN that focuses on caring for disadvantaged populations. It consists of 450 providers from 35 primary care practices that care for an underserved population, where 37% of patients are Hispanic/Latino and 44% are recipients of Medicaid or formal discount programs. The Dermatology Clinics at University of Colorado Denver and Denver Health coordinates 35,000 outpatient visits per year, and our dermatologists care for over 2500 psoriasis patients annually.

Recruiting from Northern California Psoriasis Patients:

To engage psoriasis patients from northern California, we will recruit from medically underserved areas in California as well as primary care network sites with dermatology services in northern California. Specifically, we will work with one of the largest federally qualified health centers (FQHC) in California-- the Alta Family Health Clinic in Dinuba. Located in Tulare County, Alta Family Health Clinic is the main healthcare provider for a rural population of nearly 2 million in Central California. Alta Family also serves the many migrant and seasonal farm workers who lack health insurance coverage. Other rural collaborators include Vita Dermatology in Red Bluff, which serves a rural population of 63,000 people from 19 cities in Tehama County. Designated as medically underserved areas, Tulare and Tehama counties are diverse in race and ethnicity, with over 50,000 non-whites in Tulare County and 5,000 non-whites in Tehama County. Both counties also contain substantial Hispanic residents at approximately 61% and 23% in Tulare and Tehama counties, respectively. We will also recruit psoriasis patients from student-run clinics in the Sacramento County that serve an indigent population consisting of underprivileged African Americans, Asian and Pacific Islanders, and Latinos. We will also recruit psoriasis patients from general dermatology outpatient clinics in the Placer, Sacramento, and Yolo counties.

Recruiting from Southern California Psoriasis Patients:

Enrollment of psoriasis patients from southern California will be accomplished by recruiting from a target population of approximately 99,000 adult psoriasis patients in Los Angeles County. To engage psoriasis patients from southern California, we have partnered with LA Net, the Department of Family Medicine, the Department of Dermatology at USC, LA County+USC Medical Center, the California Association of Rural Health Clinics, and California Primary Care Association.

LA Net was established in 2002 and is a well-established affiliation of Practice Based Research Networks (PBRNs) in California. LA Net members consist of a voluntary network of providers who have joined together to work to reduce health disparities. The community health resource network consists of 25 Federally Qualified Health Centers, Community Health Centers and private practices serving low-income patients representing 116 distinct practice locations in the county and providing care for more than 1.2 million patients per year. LA Net has experienced research staff dedicated to supporting the study of causes and solutions to minority health disparities in the region. We anticipate that our PCORI project will help serve the patients in LA Net, and we will be able to evaluate outcomes among minority patients with psoriasis. LA Net consists of a combination of family medicine, internal medicine, pediatrics, nursing, and other specialties. This variety of specialty and range of research interests ensure that we will be able to recruit from a diverse cohort of adult psoriasis patients.

The Department of Family Medicine at USC works closely with LA Net to develop and implement health services projects aimed at creating access and reducing disparities in

southern California. Specifically, the Center for Community Health Studies at USC focuses on research initiatives that increase access to health care among underserved populations. Furthermore, the clinics at the Department of Dermatology at USC serves as a tertiary referral center for complex psoriasis patients in southern California, and these clinics will serve as additional sites of recruitment. The Department of Dermatology is fully supportive and prepared to make this PCORI project a success.

We will also be recruiting psoriasis patients from the Los Angeles County (LAC) + University of Southern California (USC) Medical Center. LAC+USC is one of the largest public hospitals in the country. LA County hospital affiliated with the University of Southern California in 1885, establishing a long and prosperous academic partnership where the center is currently one of the premier academic teaching hospitals in the nation, and one of the state's leading hospitals for training health professionals. LAC + USC's medical staff includes more than 700 full-time physicians from the USC Keck School of Medicine, nearly 900 medical interns and residents, and 2,600 nurses. In addition to providing world-class health care, LAC + USC also works with a network of community clinics and other health care providers to coordinate hospital services and community-based care.

Finally, we have established a strong relationship with the California Primary Care Association (CPCA). CPCA is an organization that represents more than 900 nonprofit community clinics and health centers in California who provide comprehensive, quality health care services to primarily low-income uninsured and underserved Californians. Based in Sacramento and formed in 1994, the CPCA has been designated as California's primary care association and, as such, aims to assist those who face barriers to health care. The primary mission of the CPCA is to lead and position community clinics, health centers, and networks through advocacy, education and services as key players in the health care delivery system to improve the health status of their communities. CPCA's diverse membership includes community and free clinics, federally funded and federally designated clinics, rural and urban clinics, large and small clinic corporations and clinics dedicated to special needs and special populations. We anticipate that CPCA will add greatly to our project as we test the collaborative connected health model in the care of psoriasis patients in their affiliated community clinics.

We will recruit from both EHR-enabled and non-EHR enabled practices. For EHR-enabled practices, we will obtain a list of patients with psoriasis diagnosis (ICD-9 of 696.1) and then confer with practice PCPs regarding appropriate patients to contact for this study. For practices with hardcopy medical records only, we will ask PCPs and their staff to identify appropriate psoriasis patients to approach. With PCPs' permission, we will send these psoriasis patients mailing to introduce the study.

<u>Retention Strategy</u>: We will conduct pre-trial retention training and workshops for all performance-site staff and create sit-specific retention plans. We will educate all study participants regarding trial requirements and the importance of full participation during both the informed consent process and throughout the trial and provide reminders for data collection. Monetary incentives will also be used to enhance retention of subjects during the 12-month follow-up period. Upon completion of the baseline visit, each study subject will receive a $100 gift card. For each follow-up visit at month 3, 6, 9 and 12, participants will receive an additional $50 gift card for a total of $300 upon study completion. If subjects fail to complete a follow-up visit within 2 weeks of the target date, research staff will send three reminders by telephone and email. The team will make accommodations and show concern for personal situations.

4.4 Treatment Assignment Procedures

To ensure enrollment of patients representing the full spectrum of disease severity and assessment of heterogeneity of treatment effects, we will perform stratified randomization using random block sizes. This stratified randomization is based on having 1:1:2 stratification with mild (<3% BSA), moderate (3-10% BSA), and severe (>10% BSA or on phototherapy or systemic therapies) psoriasis severity.[29] Within each stratum, the patients are then randomized 1:1 to collaborative connected health or usual in-person care. Thus, these combined recruitment methods will contribute to a diverse patient population in demographic, socioeconomic, and disease states.

4.5 Subject Withdrawal

Safety is our first concern for all of our study patients. This is a pragmatic trial and therefore any real world standards currently in place are encouraged and exercised in all aspects of the study. Additionally, because this research is evaluating whether the CCH model works under real-life conditions and whether it works in terms that matter to the patient, there is no reason to remove a patient from the study. We anticipate that a small proportion of our patients will need in-person care. A participant randomized to the CCH arm who receives in-person care exclusively, or a combination of online and in-person visits will not be removed from the study. The research team will capture this occurrence on a quarterly basis with the self-reported "Access to Care" questionnaire. The frequency and reason for in-person visits will be noted in the analysis.

Subjects may withdraw voluntarily from the study at any time and their decision will have no impact on their usual care.

An investigator may terminate a subject's participation in the study if the subject meets an exclusion criterion (either newly developed or not previously recognized) that precludes further study participation.

Whenever a participant chooses to withdraw from the study or discontinue study intervention, the research team will document the specific, detailed reason for the withdrawal.

5 STUDY SCHEDULE

5.1 Informed Consent

All subjects, or legally authorized representatives, must sign an informed consent form for participation in this study prior to performing any screening procedures. Procedures listed are consistent with those included in the Schedule of Events (Table 2).

5.2 Screening Visit (Week 0)

- Review medical history to determine eligibility based on inclusion/exclusion criteria.

5.3 Enrollment/Baseline (Visit 1, Week 0)

- Verify inclusion/exclusion criteria.
- Obtain demographic information, medical history, medication history, alcohol, and tobacco use history.
- Computer Fluency Assessment
- SA-PASI
- % BSA assessment
- Quality of life questionnaires (DLQI, EG-5D-5L, Skindex-16)
- Mental health assessment (PHQ-9)
- Access to care questionnaire
- Treatment randomization
  - Psoriasis Connect instruction
  - Instructions on taking standardized pictures

5.4 Visit 2, Week 12

- Return medical history
- SA-PASI
- % BSA assessment
- Quality of life questionnaires (DLQI, EG-5D-5L, Skindex-16)
- Mental health assessment (PHQ-9)
- Access to care questionnaire

5.5 Visit 3, Week 24

- Return medical history
- SA-PASI
- % BSA assessment
- Quality of life questionnaires (DLQI, EG-5D-5L, Skindex-16)
- Mental health assessment (PHQ-9)
- Access to care questionnaire

5.6 Visit 4, Week 36

- Return medical history
- SA-PASI

- % BSA assessment
- Quality of life questionnaires (DLQI, EG-5D-5L, Skindex-16)
- Mental health assessment (PHQ-9)
- Access to care questionnaire

5.7 Visit 5, Week 48

- Return medical history
- SA-PASI
- % BSA assessment
- Quality of life questionnaires (DLQI, EG-5D-5L, Skindex-16)
- Mental health assessment (PHQ-9)
- Access to care questionnaire

Table 2: Data Collection	Baseline	3 Month	6 Month	9 Month	12 Month
Demographic, Socioeconomic, Computer-Email-Web Fluency	X				
Psoriasis severity: SA-PASI, BSA, PGA	X	X	X	X	X
Quality of life: Skindex-16, DLQI, EQ-5D-5L	X	X	X	X	X
Mental Health (Depression): PHQ-9	X	X	X	X	X
Access to care: wait time, transportation, obtaining care	X	X	X	X	X
Qualitative assessment of utility of CCH			X		X

6 ASSESSMENT OF SAFETY

6.1 Specification of Safety Parameters

6.1.1 Unanticipated Problems

The Office for Human Research Protections (OHRP) considers unanticipated problems involving risks to subjects or others to include, in general, any incident, experience, or outcome that meets all of the following criteria:

- Unexpected in terms of nature, severity, or frequency given (a) the research procedures that are described in the protocol-related documents, such as the IRB-approved research protocol and informed consent document; and (b) the characteristics of the subject population being studied;
- Related or possibly related to participation in the research ("possibly related" means there is a reasonable possibility that the incident, experience, or outcome may have been caused by the procedures involved in the research); and
- Suggests that the research places subjects or others at a greater risk of harm (including physical, psychological, economic, or social harm) than was previously known or recognized.

6.1.2 Adverse Event

An adverse event (AE) is any symptom, sign, illness or experience that develops or worsens in severity during the course of the study. Intercurrent illnesses or injuries should be regarded as adverse events. Abnormal results of diagnostic procedures are considered to be adverse events if the abnormality:

- Results in study withdrawal
- Is associated with a serious adverse event
- Is associated with clinical signs or symptoms
- Leads to additional treatment or to further diagnostic tests
- Is considered by the investigator to be of clinical significance

6.1.3 Serious Adverse Events

Adverse events are classified as serious or non-serious. A serious adverse event (SAE) is one that meets one or more of the following criteria:

- Results in death
- Is life-threatening (places the subject at immediate risk of death from the event as it occurred)
- Results in inpatient hospitalization or prolongation of existing hospitalization
- Results in a persistent or significant disability or incapacity
- Results in a congenital anomaly or birth defect
- An important medical event that may not result in death, be life threatening, or require hospitalization may be considered an SAE when, based upon appropriate medical judgment, the event may jeopardize the subject and may require medical or surgical intervention to prevent one of the outcomes listed in this definition.

6.2 Time Period and Frequency for Event Assessment and Follow-Up

Unanticipated problems will be recorded in the data collection system throughout the study.

The PI will record all reported events with start dates occurring any time after informed consent is obtained until 7 (for non-serious AEs) or 30 days (for SAEs) after the last day of study participation. If an adverse event is self-reported by the study participant, the investigator will follow the participant for outcome information until resolution or stabilization.

6.3 Characteristics of an Adverse Event

6.3.1 Relationship to Study Intervention

To assess relationship of an event to study intervention, the following guidelines are used:

1. Related (Possible, Probable, Definite)
    a. The event is known to occur with the study intervention.
    b. There is a temporal relationship between the intervention and event onset.
    c. The event abates when the intervention is discontinued.
    d. The event reappears upon a re-challenge with the intervention.
2. Not Related (Unlikely, Not Related)
    a. There is no temporal relationship between the intervention and event onset.
    b. An altemate etiology has been established.

6.3.2 Expectedness of SAEs

The Study PI will be responsible for determining whether an SAE is expected or unexpected. An adverse event will be considered unexpected if the nature, severity, or frequency of the event is not consistent with the risk information previously described for the intervention.

6.4 Reporting Procedures

All adverse events reported during the course of the study will be reported to the PI. Each week, the study investigator will review the AE Forms from the previous week for events that were reported as new or continuing. The core research team will meet on a weekly basis. The core research team, the Psoriasis Patient Advisory Panel, study collaborators, and performance sites will convene via teleconference on a monthly basis to discuss recruitment, study conduct, and new adverse events. Minutes will be taken at all research meetings for transparency and shared with among the members of the study team.

In accordance with IRB regulations, reportable adverse effects arising from any research procedures will be submitted promptly to University of Colorado Denver's Office of Human Research Protection, and the PI will be notified of any subsequent reporting by the UCD OHRP Although they are not anticipated to occur, unanticipated (non-serious) or serious adverse events in study patients will be reported to the IRB within the number of days specified by the local IRB.

7 STATISTICAL CONSIDERATIONS

7.1 Sample Size Considerations

By using the sample size formula *N* = 4e2 σ2 1/ 4(1+3*rho)/ *δ12* with e = *Z1-α* + *Z1-β/2,* where *Zp* is the *p* percentile of the standard normal distribution, *δ1= δU* - |*δ*| at a significance level of α = 0.05, we calculated that, with a total of 254 participants (127 per each arm), the study will achieve a power of 75-99% depending on σ and rho (ρ). Note that the power only drops below 80% for σ ≈ 20% and rho (ρ) ≈ 0.8. We will enroll a total of 300 patients (150 per each arm) by considering a dropout rate of 15%.[1,34] Thus, all power estimates are conservative because we will have partial information on the 45 patients who dropout.

7.2 Statistical Methods

7.2.1 Aim 1: Psoriasis Disease Severity Outcomes

SAS 9.3 will be used for analysis. All data will be summarized using descriptive statistics (mean, SD, range for continuous variables; frequencies for categorical variables) and graphical techniques. Tables will be produced describing any missing data patterns due to either withdrawal or other reasons. In this equivalency trial, we will use an intention-to-treat (ITT) approach to analyze all primary and secondary outcomes.

For the primary outcome (Aim 1), the mean percent improvement in SA-PASI will be averaged over 3, 6, 9, and 12 months. The percent improvement in SA-PASI is defined as the difference in SA-PASI scores between the baseline and each follow-up visits divided by the SA-PASI score from the baseline visit. We chose the mean across the four follow-up assessments for two reasons: (1) it would be sensitive to early improvements as well as later benefits, and (2) it is statistically more efficient than an endpoint based on a single assessment.

Change in SA-PASI will be analyzed longitudinally, using repeated measures regression (Diggle, 2002), to test the equivalence hypothesis of no difference between arm specific estimates. Let μC and μI be the expected change in PASI in the control arm and intervention arm, respectively. Let δ = μI - μC. The goal is to establish that $\delta_L < \delta < \delta_U$, where δ_L and δ_U, are a priori specified values used to define equivalence. In practice, δ_L and δ_U are often chosen such that $\delta_L = -\delta_U$. The null hypothesis H0: $\delta \le \delta_L$ or $\delta \ge \delta_U$ is tested against the two-sided alternative hypothesis Ha: $\delta_L < \delta < \delta_U$, at significance level α = 0.05. We present a range σ and ρ assumptions and power calculations in Table 3.

Table 3. Psoriasis severity outcome measure statistics

Measure	Standard Deviation (σ)	Correlation rho (ρ)	Equivalence Margin	Power
SA-PASI	15%-20%	0.5-0.8	+/- 6.5	0.75-0.99
BSA	18% (16%-20%)	0.5-0.8	+/- 6.5	0.75-0.99

PGA	0.6 to 0.7	0.5-0.8	+/- 0.25	0.84-0.99

We consider $\delta U = \pm 6.5$ as the equivalence limit, which is half of a clinically significant difference and more conservative than prior equivalency studies with equivalency limits between 10-20%.[1,45] Assume the absolute mean difference of the change in SA-PASI $|\delta|=0$ between the two arms and that the standard deviation of the change in SA-PASI $\sigma C = \sigma I = \sigma = 15$ -20%. We expect the correlation (rho) of the four follow-up assessments to range between 0.5 and 0.8.

To assess whether CCH may have different effects on patients with varying psoriasis severity, we have incorporated into the study design stratified randomization *a priori* to address potential heterogeneity of treatment effects. Specifically, the stratified randomization is based on having 1:1:2 stratification of mild (<3% BSA), moderate (3- 10% BSA), and severe (>10% BSA or on phototherapy or systemic therapy) psoriasis severity.[29] Within each stratum, the patients are then randomized 1:1 to collaborative connected health or usual in-person care. Thus, within each stratum of psoriasis severity, the patients will have balanced baseline characteristics between the two intervention arms. To test for heterogeneity of treatment effects (HTE), an interaction term between severity and treatment group will be introduced to the model. If the interaction term is significant, then contrasts between each group (severity by treatment) will be computed to identify the source of HTE.

7.2.2 *Aim 2: Dermatology-Specific, Quality-of-Life Outcomes and Mental Health Outcomes*

Quality-of-life assessments are critical in the evaluation of novel, technology-enabled healthcare delivery models. We have obtained permissions to use dermatology-specific quality-of-life instruments Skindex-16 and Dermatology Life Quality Index (DLQI) for this proposed study.[35] Skindex-16 is a validated and reliable instrument that comprehensively captures the effects of skin disease on health-related quality of life.[36] It discriminates among patients with different effects and is responsive to clinical changes over time.[37] Skindex-16 scores range from 0 (no effect) to 100 (effect experienced all the time), and the responses are aggregated in symptoms, emotions, and functioning scales. We will also use DLQI, another validated dermatology-specific quality-of-life instrument that has been used in many psoriasis trials. DLQI scores range from 0 to 30, with higher scores indicating more severe impact on quality of life.[11] Because the psychometric properties of the two instruments differ in some aspects, using both instruments enables comparison of study findings with previous work in dermatology.[38]

The Patient Health Questionnaire (PHQ) is a validated, self-administered version of PRIME-MD diagnostic instrument for common mental disorders.[39] The PHQ-9 is the depression module, which scores each of the 0 DSM-IV criteria as "0" (not at all) to "3" (nearly every day). A PHQ-9 score of 10 or greater has 89% sensitivity and 88% specificity for major depression. PHQ-9 scores of 5, 10, 15, and 20 represented mild, moderate, moderately severe, and severe depression, respectively. PHQ-9 is a validated tool for diagnosis of depression and monitoring response to interventions.

The same repeated-measures, equivalency-evaluation approach described in Aim 1 will be used to compare Skindex-16, DLQI and PHQ-9 scores between patients randomized to the CCH model and in-person care. Effect sizes of approximately 0.5 of the standard deviation are generally considered to be clinically significant for QOL measures. We have specified equivalence margins that exclude effect sizes of that magnitude (Table 4).

Table 4. Quality of life and mental health outcomes measures equivalency margins				
Measure	Standard Deviation (σ)	Rho (ρ)	Equivalence Margin	Power
DLQI	7 (6.5-8)	0.5-0.8	+/- 2.5	0.70-0.99
Skindex-16	20	0.5-0.8	+/- 7	0.83-0.94
PHQ-9	8 (7-10)	0.5-0.8	+/-3.0	0.82-0.99

7.2.3 *Aim 3a: Assess Access-to-Specialist Care and Analyze Access-to-Care Measures*

To determine whether CCH provides better access to specialist care compared to the in-person model, we will ask patients to complete access-to-care questions derived from the Medical Expenditure Panel Survey (MEPS) and Medicare Current Beneficiary Survey (MCBS) once every 3 months.[40] We will confirm their responses with chart review.

Specifically, we will use the following access-to-specialist-care measures: transportation factors and wait time and difficulties associated with obtaining specialist are.[41,42] Transportation factors and wait time will be evaluated along the following three dimensions:[42] (a) total distance travelled to see a dermatologist as defined by the round-trip distance from a patient's home to the dermatologist's office multiplied by the number of in-person visits during the 12-month period, (b) total transportation time as defined by the round-trip transit time multiplied by the number of in-person visits to a dermatologist's office, and (c) transportation mode as categorized into walking, driving using one's own car, or taking taxi or public transportation. While transportation factors pertain mostly to patients in the in-person arm, some patients in the CCH model may require in-person specialist evaluation during the study. Difficulties associated with obtaining specialist care will be assessed based on questions and scales derived from the MEPS and MCBS. We will ask patients the following questions: "How difficult is it to see a dermatologist in-person/online initially?" "How difficult is it to contact the dermatologist about a skin problem?" "How difficult is it to get an online/in-person appointment with a dermatologist on short notice?" The response choices derived from MEPS are on an ordinal scale.[42]

The hypothesis for Aim 3A is that the collaborative connected health model will provide superior access to specialist care than in-person model. Based on an estimated 254 participants completing the trial (after accounting for a 15% dropout rate) and significance level of 0.05, we present the power calculations and analytical methods for the measures in Aim 3A in Table 6.

Table 6. Access to care outcome measures statistics

Measures	Variable Type	Std. Dev.	Effect Size	Power	Analytical Method
Total distance travelled to see a specialist per year	Continuous (miles)	CCH: 5-15<br>IP: 15-30	60 miles	>99%	Linear Regression
Total transportation time to see a specialist per year	Continuous (minutes)	CCH: 15-45<br>IP: 45-70	180 minutes	>99%	Linear Regression
Transportation mode	Nominal	Not applicable			Descriptive statistics
Difficulties associated with obtaining specialist care	Ordinal	Not applicable	OR: 0.5-0.55	92%-99%	Logistic regression

We will perform qualitative data collection and analysis to obtain patient and clinician perspectives on the utility of CCH for increasing specialty-care access. Specifically, we will conduct semi-structured interviews using an interview guide that allows for flexibility in exploring the emerging themes. The interviewer will take field notes on a standard assessment sheet. The interviews will also be audio recorded for later review, clarification, and analysis. A brief case summary incorporating key findings will be completed within 24 hours of the interview. This will be reviewed by the PI to identify any areas of disagreement or uncertainty about the interpretation of findings. The interviews will be conducted at 6 months and then repeated at 12 months with the same participants.

We will employ qualitative analytical techniques with investigator triangulation and member checking to enhance the validity of the conclusions drawn. Specifically, once the data have been collected from the various stakeholders, a template style of analysis will be used to organize the data for reflection and development of emerging themes.[141,142] The initial list of codes will be based on broad thematic areas we expect to see in the data based on our questions. Template coding also allows for using additional codes for emerging themes analysts observe early in the coding. Coded data will be further reviewed to refine and develop provisional themes for further reflection. Drs. Armstrong, West, Gelfand, and Westfall will then reflect on the data together to identify contrasts and commonalities and to derive final conclusions.

7.3 Addressing Missing Data

In this section, we describe methods to prevent, monitor, report, and handle missing data for all 3 specific aims of the study.

7.3.1 Prevent, Monitor, and Report Missing Data

We will conduct pre-trial retention training and workshops for all performance-site staff and create site-specific retention plans. We will educate all study participants regarding trial

requirements and the importance of full participation during both the informed consent process and throughout the trial and provide reminders for data collection. From the pilot study, our analysis did not reveal particular patient characteristics that were significantly associated with certain types of missing data. Finally, to monitor missing data, we have created case report forms containing structured fields that seek reasons for non-reporting or for subject withdrawal; these structured fields included forgetting to report outcomes at particular time intervals, perceived lack of efficacy of the model, and inability to comply with study requirements. We also have write-in fields for other potential reasons for missing data.

7.3.2 Statistical Methods to Handle Missing Data and Sensitivity Analyses

We will use Mixed Models for Repeated Measures (MMRM) as the primary analysis for handling missing data. The MMRM model assumes missing at random (MAR), which is most appropriate for studies where the primary objective is to assess equivalence,[44] as is in this study. While MAR's assumption may not be applicable for all subjects, the use of MAR is appropriate because there is no reason to suspect that study factors or differential outcomes will drive subject dropout.

We will perform sensitivity analyses to assess the robustness of the primary estimate with regards to missing data. Specifically, we will use the missing-not-at-random (MNAR) model to test the possibility that the missing data points are not random and are informative to the study hypothesis.

In the case of access-to-care measures, the models are not longitudinal in nature, and each subject has, effectively, one measurement data point. Subjects who do not have complete data for these variables will be excluded from these models.

8 PATIENT AND STAKEHOLDER ENGAGEMENT

In this section, we will expand on the nature, degree, and process of patient and stakeholder engagement during the development of the research plan and in the key aspects of the proposed project.

8.1 Patient and Stakeholder Engagement in Monitoring Study Conduct and Progress

During the study period, we will conduct monthly conference calls with the stakeholders to discuss the study progress and to identify areas of improvement. The members of the Psoriasis Patient Advisory Council will work with the core research staff to monitor the study progress via additional evaluations of study progress. Specifically, the Council members will be provided with new feedback from the study participants since the last progress update. The Council members will evaluate the ongoing and new patient feedback and advise the core research team on patient engagement, protection, and continued enhancement of patient experiences throughout the study. This also enables the research staff to address any patient concerns expediently.

8.2 Patient and Stakeholder Engagement in the Dissemination of Research Results

We anticipate that the study results will be disseminated rapidly and widely with the engagement of patients and stakeholders. The National Psoriasis Foundation and the American Academy of Dermatology are committed to disseminate these findings to their active patient constituency via multiple media formats. Through collaborations with these two organizations, our study team has the capability to involve 85,000 engaged psoriasis patients in the U.S. for dissemination of these study findings. These 85,000 engaged psoriasis patients can help reach the nearly 10 million psoriasis patients in the U.S via mass media communication strategies used by the NPF and AAD. Furthermore, the Center for Connected Health Policy will disseminate the study findings in the health policy communities through publishing white papers and drafting new telehealth legislations based on the study findings. In addition to supporting our manuscript submission efforts to the highest-tier journals, the AAD and the American Telemedicine Association are also prepared to disseminate the research results via JAMA Dermatology, Journal of the American Academy of Dermatology, and Telemedicine and eHealth.

Moreover, the research team is attending the California Association of Rural Health Clinic's (CARHC) 8th annual conference for their member rural health centers. At the conference, we will present our PCORI project to inform California Rural Health Clinics about our ongoing research in teledermatology. This opportunity to share our project with rural clinics will likely cultivate relationships and build interest and anticipation about future study results. CARHC is eager and committed to disseminate our study findings at these annual forums and act as a conduit to distribute relevant findings to member health centers.

Upon completion of the study, the research team will also convene with the Department of Family Medicine at USC to discuss the findings and identify further opportunities for dissemination. If the results of the pragmatic trial are successful, physicians at USC could devise ways of scaling this CCH model so that it may be implemented within USC's primary care system.

8.3 Shared Decision-Making and PCORI Engagement Principles

Our research team is committed to reciprocal relationships, co-learning between the research team and patient partners, valued partnership, and research that is grounded in trust, transparency, and honesty. We will engage patient partners in monitoring patient-centeredness during the study. We will meet regularly with our patient partners and other stakeholders to review study progress. We make important study-related decisions through engaging all stakeholders and will arrive at final decisions through consensus. We will also document all research meetings via minutes for transparency and share them with our research partners.

9 ETHICS/PROTECTION OF HUMAN SUBJECTS

9.1 Ethical Standard

The investigator will ensure that this study is conducted in full conformity with the principles set forth in The Belmont Report: Ethical Principles and Guidelines for the Protection of Human Subjects of Research, as drafted by the US National Commission for the Protection of Human Subjects of Biomedical and Behavioral Research (April 18, 1979) and codified in 45 CFR Part 46 and/or the ICH E6.

9.2 Institutional Review Board

The protocol, informed consent form(s), recruitment materials, and all subject materials will be submitted to the IRB for review and approval. Approval of both the protocol and the consent form must be obtained before any subject is enrolled. Any amendment to the protocol will require review and approval by the IRB before the changes are implemented in the study.

9.3 Informed Consent Process

Informed consent is a process that is initiated prior to the individual agreeing to participate in the study and continues throughout study participation. Extensive discussion of risks and possible benefits of study participation will be provided to subjects and their families, if applicable. A consent form describing in detail the study procedures and risks will be given to the subject. Consent forms will be IRB-approved, and the subject is required to read and review the document or have the document read to him or her. Only trained study staff listed on the protocol will obtain by explaining the research study to the subject and answer any questions that may arise. The subject will sign the informed consent document prior to any study-related assessments or procedures. Subjects will be given the opportunity to discuss the study with their surrogates or think about it prior to agreeing to participate. They may withdraw consent at any time throughout the course of the study. A copy of the signed informed consent document will be given to subjects for their records. The rights and welfare of the subjects will be protected by emphasizing to them that the quality of their clinical care will not be adversely affected if they decline to participate in this study.

9.4 Subject Confidentiality

Information about study subjects will be kept confidential and managed according to the requirements of the Health Insurance Portability and Accountability Act of 1996 (HIPAA). Those regulations require a signed subject authorization informing the subject of the following:

- What protected health information PHI will be collected from subjects in this study
- Who will have access to that information
- Who will use or disclose that information
- The rights of a research subject to revoke their authorization for use of their PHI

In the event that a subject revokes authorization to collect or use PHI, the investigator, by regulation, retains the ability to use all information collected prior to the revocation of subject authorization. For subjects that have revoked authorization to collect or use PHI, attempts should be made to obtain permission to collect at least vital status (i.e. that the subject is alive) at the end of their scheduled study period.

Subject confidentiality is strictly held in trust by the investigators, study staff, and the sponsor(s) and their agents. The study protocol, documentation, data, and all other information generated will be held in strict confidence. No information concerning the study or the data will be released to any unauthorized third party without prior written approval of the sponsor.

The study monitor or other authorized representatives of the sponsor may inspect all study documents and records required to be maintained by the investigator, including but not limited to, medical records (office, clinic, or hospital) for the study subjects. The clinical study site will permit access to such records.

10 DATA HANDLING AND RECORD KEEPING

The investigators are responsible for ensuring the accuracy, completeness, legibility, and timeliness of the data reported.

10.1 Data Management Responsibilities

Data collection and accurate documentation are the responsibility of the study staff under the supervision of the investigator. All source documents must be reviewed by the study team and data entry staff, which will ensure that they are accurate and complete. Unanticipated problems and adverse events must be reviewed by the investigator or designee.

10.2 Data Capture Methods

All data will be collected and stored in a password-protected, HIPAA-compliant database called Research Electronic Data Capture (REDCap) that only the investigators, study staff, IRB, and other authorized/designated individuals will be allowed access. All HIPAA, IRB, State and Federal policies and guidelines will be followed in ensuring confidentiality. The PI will review safety parameters prior to enrollment. Patient survey data will be securely stored on REDCap for five years after the completion of the study, as required by the project sponsor. During the five years for which the records are retained, only study subject personnel will have access to the password-protected data.

The PI and study personnel will evaluate the online teledermatology platform, Psoriasis Connect, on a monthly basis to ensure that all security features of the teledermatology platform continue to adhere to the highest security standards and that patient information are kept secure. Photographs uploaded onto the teledermatology platform will be removed from the database and destroyed immediately upon subject's completion of the study. The PI and study staff will also convene with the Psoriasis Patient Advisory Panel to address any concerns that the study patients have regarding study procedures and/or personal privacy issues with the platform.

10.3 Protocol Deviations

A protocol deviation is any noncompliance with the clinical study protocol, Good Clinical Practice, or Manual of Procedures requirements. The noncompliance may be on the part of the subject, the investigator, or study staff. All deviations from the protocol must be addressed in study subject source documents and promptly reported to PCORI and the local IRB, according to their requirements.

11 PUBLICATION/DATA SHARING POLICY

The principal investigator will register the trial in ClinicalTrials.gov. In accordance with PCORI's legislative mandate, the research team will collaborate with PCORI in order for PCORI to make the PCORI-funded research findings available to clinicians, patients and the general public not later than ninety days after the conduct or receipt of research findings. Prior to public dissemination of any research findings, the team will coordinate the publication, press release, website posting or public email announcement with PCORI at least thirty days in advance. Additionally, the research team will submit to the PCORI manager via email all accepted presentations and full-length peer-reviewed publications related to the research, notify PCORI annually all peer review rejections and report all plans for additional communications to PCORI for five years post-research project completion.

12 LITERATURE REFERENCES

1. Chambers CJ PK, Schupp C, and Armstrong AW. Patient-Centered Online Management of Psoriasis: A Randomized Controlled Equivalency Trial. 2011.

2. Ramsay DL, Weary PE. Primary care in dermatology: whose role should it be? Journal of the American Academy of Dermatology. Dec 1996;35(6):1005-1008.

3. Federman D, Hogan D, Taylor JR, Caralis P, Kirsner RS. A comparison of diagnosis, evaluation, and treatment of patients with dermatologic disorders. Journal of the American Academy of Dermatology. May 1995;32(5 Pt 1):726-729.

4. Fleischer AB, Jr., Feldman SR, Rapp SR. Introduction. The magnitude of skin disease in the United States. Dermatol Clin. Apr 2000;18(2):xv-xxi.

5. Bystryn JC. Dermatology manpower needs. Dermatol Clin. Apr 2000;18(2):303-312, x.

6. Craiglow BG, Resneck JS, Jr., Lucky AW, et al. Pediatric dermatology workforce shortage: perspectives from academia. J Am Acad Dermatol. Dec 2008;59(6):986-989.

7. Kimball AB, Resneck JS, Jr. The US dermatology workforce: a specialty remains in shortage. J Am Acad Dermatol. Nov 2008;59(5):741-745.

8. Resneck J, Jr., Kimball AB. The dermatology workforce shortage. J Am Acad Dermatol. Jan 2004;50(1):50-54.

9. Van Voorhees AS, Fried R. Depression and quality of life in psoriasis. Postgrad Med. Jul 2009;121(4):154-161.

10. Rachadonda TD, Schupp CW, Armstrong AW. Psoriasis Prevalence among adults in the United States. J Am Acad Dermatol. 2013;in press.K

11. Kurd SK, Gelfand JM. The prevalence of previously diagnosed and undiagnosed psoriasis in US adults: results from NHANES 2003-2004. J Am Acad Dermatol. Feb 2009;60(2):218-224.

12. Bailey EE, Ference EH, Alikhan A, Hession MT, Armstrong AW. Combination treatments for psoriasis: a systematic review and meta-analysis. Archives of dermatology. Apr 2012;148(4):511-522.

13. Naldi L, Mercuri SR. Epidemiology of comorbidities in psoriasis. Dermatologic therapy. Mar-Apr 2010;23(2):114-118.

14. Haddad A, Chandran V. Arthritis mutilans. Current rheumatology reports. Apr 2013;15(4):321.

15. Armstrong AW, Harskamp CT, Armstrong EJ. Psoriasis and metabolic syndrome: a systematic review and meta-analysis of observational studies. J Am Acad Dermatol. Apr 2013;68(4):654-662.

16. Riemsma R, Al M, Ramos IC, et al. SeHCAT tauroselcholic (selenium-75) acid for the investigation of bile acid malabsorption and measurement of bile acid pool loss: a systematic review and cost-effectiveness analysis. Health Technology Assessment. Dec 2013;17(61):1-+.

17. Armstrong EJ, Harskamp CT, Armstrong AW. Psoriasis and major adverse cardiovascular events: a systematic review and meta-analysis of observational studies. Journal of the American Heart Association. Apr 2013;2(2):e000062.

18. Gelfand JM, Neimann AL, Shin DB, Wang X, Margolis DJ, Troxel AB. Risk of myocardial infarction in patients with psoriasis. JAMA : the journal of the American Medical Association. Oct 11 2006;296(14):1735-1741.

19. American Telemedicine Association: What is Telemedicine? 2013; http://www.americantelemed.org/learn. Accessed 12-31, 2013.

20. Ekeland AG, Bowes A, Flottorp S. Effectiveness of telemedicine: a systematic review of reviews. Int J Med Inform. Nov 2010;79(11):736-771.

21. Eminovic N, de Keizer NF, Bindels PJ, Hasman A. Maturity of teledermatology evaluation research: a systematic literature review. Br J Dermatol. Mar 2007;156(3):412-419.

22. Whited JD. Teledermatology research review. Int J Dermatol. Mar 2006;45(3):220-229.

23. Collins K, Walters S, Bowns I. Patient satisfaction with teledermatology: quantitative and qualitative results from a randomized controlled trial. J Telemed Telecare. 2004;10(1):29-33.

24. Krupinski E, Barker G, Rodriguez G, et al. Telemedicine versus in-person dermatology referrals: an analysis of case complexity. Telemed J E Health. Summer 2002;8(2):143-147.

25. Hsu L, Snodgrass BT, Armstrong AW. Anti-drug Antibodies in Psoriasis: A Systematic Review. Br J Dermatol. Oct 1 2013.

26. Parsi KK CC, and Armstrong AW. Cost-Effectiveness Analysis of a Patient-Centered Online Care Model for Management of Psoriasis. 2011.

27. Thorpe KE, Zwarenstein M, Oxman AD, et al. A pragmatic-explanatory continuum indicator summary (PRECIS): a tool to help trial designers. Journal of clinical epidemiology. May 2009;62(5):464-475.

28. Zwarenstein M, Treweek S, Gagnier JJ, et al. Improving the reporting of pragmatic trials: an extension of the CONSORT statement. Bmj. 2008;337:a2390.

29. Pariser DM, Bagel J, Gelfand JM, et al. National Psoriasis Foundation clinical consensus on disease severity. Archives of dermatology. Feb 2007;143(2):239-242.

30. Feldman SR, Clark AR, Venkat AP, Fleischer AB, Jr., Anderson RT, Rajagopalan R. The Self- Administered Psoriasis Area and Severity Index provides an objective measure of psoriasis severity. Br J Dermatol. Feb 2005;152(2):382-383.

31. Fleischer AB, Jr., Feldman SR, Dekle CL. The SAPASI is valid and responsive to psoriasis disease severity changes in a multi-center clinical trial. The Journal of dermatology. Apr 1999;26(4):210-215.

32. Ramsay B, Lawrence CM. Measurement of involved surface area in patients with psoriasis. The British journal of dermatology. Jun 1991;124(6):565-570.

33. Leonardi CL, Powers JL, Matheson RT, et al. Etanercept as monotherapy in patients with psoriasis. N Engl J Med. Nov 20 2003;349(21):2014-2022.

34. Armstrong AW. Patient-Centered Online Care Model for Follow-Up Management of Atopic Dermatitis.manuscript in preparation.

35. SKINDEX questionnaire-16 and 29-items version. 2013; http://www.mapi-trust.org/services/questionnairelicensing/catalog-questionnaires/299-skindex. Accessed 12-18, 2013.

36. Chren MM, Lasek RJ, Quinn LM, Mostow EN, Zyzanski SJ. Skindex, a quality-of-life measure for patients with skin disease: reliability, validity, and responsiveness. The Journal of investigative dermatology. Nov 1996;107(5):707-713.

37. Chren MM, Lasek RJ, Sahay AP, Sands LP. Measurement properties of Skindex-16: a brief quality-of-life measure for patients with skin diseases. Journal of cutaneous medicine and surgery. Mar-Apr 2001;5(2):105-110.

38. Finlay AY, Khan GK. Dermatology Life Quality Index (DLQI)--a simple practical measure for routine clinical use. Clin Exp Dermatol. May 1994;19(3):210-216.

39. Kroenke K, Spitzer RL, Williams JB. The PHQ-9: validity of a brief depression severity measure. Journal of general internal medicine. Sep 2001;16(9):606-613.

40. National Healthcare Disparities Report 2012. 2012; http://www.ahrq.gov/research/findings/nhqrdr/nhdr12/nhdr12_prov.pdf. Accessed 12-27, 2013.

41. Agency for Healthcare Research and Quality: Access to Care Measures. 2013; http://www.ahrq.gov/research/findings/nhqrdr/nhdr02/premeasurea.html. Accessed 12-27, 2013.

42. MEPS Access to Care Supplement - P15R5/P16R3/P17R1. 2013; http://meps.ahrq.gov/survey_comp/hc_survey/2011/AC110311.pdf. Accessed 12-27, 2013.

43. Crabtree BF, Miller WL. Doing Qualitative Research. Thousand Oaks, CA: Sage Publications; 1999.

44. Dillman DA, Smyth JD, Christian LM. Internet, Mail, and Mixed-Mode Surveys: The Tailored Design Method. 3 ed. Hoboken, NJ: Wiley; 2009.

45. Griffiths CE, Strober BE, van de Kerkhof P, et al. Comparison of ustekinumab and etanercept for moderate-to-severe psoriasis. N Engl J Med. Jan 14 2010;362(2):118-128.

46. Armstrong AW. Agency for Healthcare Quality and Research (AHRQ) K08 Mentored Clinical Scientist Research Career Development Award (1K08HS018341-01): Patient-Centered Online Care Model for Follow-Up Management of Atopic Dermatitis.

47. Enders C. Applied Missing Data Analysis: Methodology in the Social Sciences. 1st ed: Guilford Press; 2010.
